# The impact of COVID-19 on menstrual cycle’s alterations, in relation to depression and sleep disturbances: a prospective observational study in a population of medical students

**DOI:** 10.1186/s12905-024-02971-x

**Published:** 2024-02-19

**Authors:** Daniela Polese, Flavia Costanzi, Paola Bianchi, Antonio Frega, Filippo Bellati, Maria Paola De Marco, Pasquale Parisi, Oliviero Bruni, Donatella Caserta, Giuliana Cozza

**Affiliations:** 1 PhD Program on Sensorineural Plasticity, Department of Neuroscience, Mental Health and Sensory Organs NESMOS, Sapienza University of Rome, Sant’Andrea University Hospital, Via di Grottarossa 1035-1039, Rome, 00189 Italy; 2 Gynecology Division, Department of Medical and Surgical Sciences and Translational Medicine, Sant’Andrea University Hospital, Sapienza University of Rome, Via di Grottarossa 1035-1039, Rome, 00189 Italy; 3 Chair of Pediatrics, NESMOS Department, Faculty of Medicine & Psychology, Sapienza University c/o Sant’Andrea University Hospital, Via di Grottarossa 1035-1039, Rome, 00189 Italy; 4 Department of Social and Developmental Psychology, Sapienza University, Via dei Marsi 78, Rome, 00185 Italy

**Keywords:** Sars-cov2, Amenorrhea, Dysmenorrhea, Premenstrual syndrome, Psychological stress, Post-traumatic stress disorder

## Abstract

**Background:**

The sars-Cov-2 pandemic has determined psychological stress, particularly in the young population of medical students. We studied the impact of the pandemic on menstrual cycle alteration in relation to psychological stress, presence of depression, sleep disturbances and post-traumatic stress, on a population of medical students.

**Methods:**

293 female students at the Faculty of Medicine and Psychology of the Sapienza University of Rome (23.08 years old ± 3.8) were enrolled. In March 2021, one year after quarantine, a personal data sheet on menstrual cycle, examining the quality of the menstrual cycle during the pandemic, compared to the previous period. Concomitantly, the Beck Depression Inventory and the Impact of Event Scale have been administered. A Pearson chi-square test was assessed to evaluate the difference between the characteristics of the menstrual cycle and the scores obtained with the questionnaires.

**Results:**

A statistically significant association between menstrual alterations and stress during pandemic had been found. The onset of depressive symptoms and sleep disturbances was observed in 57.1% and in 58.1% of young women with cycle’s alterations, respectively. Amenorrhea was three times more common in female students with depressive symptoms, premenstrual syndrome had a significant correlation with *both* depression and sleep disturbances. The pandemic has been related to menstrual alterations, with depressive symptoms and sleep disorders. Amenorrhea is connected to depression, as observed on the functional hypothalamic amenorrhea.

**Conclusions:**

The pandemic affected the menstrual cycle as well as the depressive symptoms and sleep. Practical implications of the study lead to the development of strategies for psychological intervention during the pandemic experience, in order to help medical trainees, with specific attention to women’s needs. Future studies should analyze the impact of other types of social stress events, on sleep, depression and the menstrual cycle beside the pandemic.

**Supplementary Information:**

The online version contains supplementary material available at 10.1186/s12905-024-02971-x.

## Introduction

The COVID-19 pandemic has changed countless aspects of life around the world [1]. The essential modus of prevention from COVID-19 infection has been isolation, social distancing strategies, environmental ventilation and disinfection [2, 3]. Various countries have started containment measures or lockdowns, closing schools, universities, commercial activities, and activities. In Italy, at the beginning of pandemic, the children and their families lived in isolation for two months until 3 May 2020, and schools and universities remained closed until September. Thus, more than 90% of young people had to leave frontal teaching activities. These circumstances were beyond everyday experience, and it has been shown how they have led to stress, anxiety and a feeling of helplessness [4, 5].

Studies reported that, already in usual conditions, university students can feel hopeless, depressed, even suicidal, and several interventions have been studying to help doctors in training and to prevent mental illness and its consequences in this population [6, 7].

Moreover, the UK General Medical Council reported a 50% rise in Mental Health issues in UK medical students [8]. Research suggested this mental health deterioration during medical school years continues when trainees enter the workforce [9].

The pandemic had a very high impact on students’ daily life. In particular, medical students have been strictly in contact with the beginning of COVID-19 Public Health Emergency of International Concern [10], especially if they are involved with the event as trainees, like medical students, with particular reactions in female population. Then they were relieved from clinical activities and started studying on digital platforms only [11, 12]. All these changes linked to the pandemic determined a strong stressor, profoundly influencing their psychological well-being [13] mediated by the activation of the hypothalamic–pituitary–adrenal axis. High cortisol levels due to psychological stress can afflict several body functions, including the menstrual cycle. In particular, psychological stress is a known risk factor for developing functional hypothalamic amenorrhea and menstrual cycle alterations [14–16], as well as psychological disorders and personality characteristics [17]. Several studies have shown that high cortisol levels due to stress are often associated with sleep disturbances, depression [18, 19] and menstrual irregularities [20, 21].

Unfortunately, questions about menstruation have been excluded from most large-scale COVID-19 studies, and the connection between fluctuations in menstrual features and the stress that the pandemic has provoked is still unclear. Only a few studies reported that women have experienced changes in their menstrual cycle during a pandemic [22–24]. These were either due to pandemic-related factors, such as behavioural restrictions, COVID-19 infection, vaccine, treatment, and/or due to the illness itself [25–27].

In addition, a recent study on female students in Jordan has highlighted that pandemic-related psychological distress has been closely linked to menstrual, premenstrual, and genitourinary symptoms [28]. The same authors have reported how the pandemic stress had an impact on menstruation changes in medical students [29]. Since the specific correlation between the menstrual cycle, psychological disorders and sleep during the pandemic in young adults has never been reported, the present study aims to identify the impact of COVID-19 on the variations in the menstrual cycle in a young population of female medical students in Rome (Lazio, Italy), analysing the menstrual cycles’ alterations and their connection with stress symptoms, as well as a post-traumatic stress disorder, depression and sleep disturbances.

## Methods

A total of 293 female students at the Faculty of Medicine and Psychology of the Sapienza University of Rome have been enrolled in the study. Students included in the study were young adults (average age 23.08 years old ± 3.8), and all were subjected to quarantine from March 2020 to June 2020. The characteristics of the population are shown in Table 1.

**Table 1:** Characteristics of study population

Characteristic	Students (n 293)
Age	22 (19–25)
Country	
Europe Africa Asia America	286 2 1 4
BMI	22.3 (19.4–26.3)
Parity	
0 > 1	279 14

Two internationally validated assessment scales, such as the Impact of Event Scale and Beck Depression Inventory, and a personal data sheet with particular relevance to the menstrual cycle and any changes in body weight and physical exercise, were administered in March 2021.

The Impact of Event Scale is a standardized psychometric scale comprising 22 items to investigate the presence of post-traumatic symptoms. This instrument comprises three sub-dimensions (Re-experience, Avoidance and Hyperarousal, including Sleep disturbances). Respondents must rate each item on a scale from 0 (not at all) to 4 (extremely) based on their experience concerning the traumatic event. Impact of Event Scale is a practical assessment for quantifying stress reactions after traumatic events. It is a valuable tool for identifying individuals who present post-traumatic symptoms and would require specialist intervention [30]. In the statistical analysis, the value of 33 has been used as a cut-off out of a maximum score of 88, considering a value greater than 33 associated with the likelihood of suffering from post-traumatic stress disorder [31].

Beck Depression Inventory (BDI) has been developed from clinical observations of attitudes and symptoms frequently occurring in depressed patients [32]. BDI is a self-assessment tool consisting of 21 multiple-choice items, and it measures the severity of depression from 13 years of age. The 21 items reflect symptoms and attitudes commonly found among clinically depressed individuals (e.g., Mood disorders, Sleep disturbances, Self-dislike). In the statistical analysis, the value of 15 has been used as a cut-off, considering that a value greater than 15 can be associated with the possible presence of depression [33].

The personal sheet, which was administered to the students, consisted of a multiple-choice questionnaire of 11 questions that investigated the general characteristics of female students, such as age, weight, height and the menstrual cycle characteristic in the last year (Table 2). In particular, the questionnaire examined the quality of the menstrual cycle during the pandemic, compared to the previous period: changes in duration, a reduction or an increase of the interval between menstrual cycles (polymenorrhea or oligomenorrhea) or the absence of period (amenorrhea). Moreover, the presence of pain during the cycle (dysmenorrhea) has also been explored. The questionnaire also examined changes in body weight and physical activity performed last year.

**Table 2:** Profile of menstrual cycle from pandemic

	All students
	N (%) 293 (100%)
Are you sexually active?	
NO YES	88 (30%) 205 (70%)
Have you noticed any changes in your menstrual rhythm in the last year, from pandemic?	
NONE INCREASE OF INTERVAL DECREASE OF INTERVAL	183 (62.5%) 73 (24.9%) 37 (12.6%)
How often have you had your period in the last year, from pandemic?	
21–35 GG > 35 < 21	260 (80.7%) 29 (9.9%) 4 (1.4%)
Have you had bouts of amenorrhea in the last year, from pandemic?	
NO YES	270 (92.2%) 23 (7.8%)
Do you suffer from dysmenorrhea?	
NO YES, IT IS INCREASED IN THE LAST YEAR, FROM PANDEMIC YES, IT IS DECREASED IN THE LAST YEAR, FROM PANDEMIC NO ALTERATION IN THE LAST YEAR, FROM PANDEMIC	185(63.1%) 37 (12.6%) 12 (4.1%) 59 (20.1%)
How do you consider the amount of your menstrual cycle?	
NORMAL ABUNDANT RARE	175 (59.7%) 78 (26.6%) 40 (13.7%)
Have you noticed any changes in the amount of your menstrual cycle in the last year, from pandemic?	
NORMAL ABUNDANT RARE	189 (64.5%) 42 (14.3%) 62 (21.2%)
Have you noticed any changes in your body weight in the last year, from pandemic?	
NO INCREASE DECREASE	132 (45.1%) 86 (29.4%) 75 (25.6%)
Have you noticed the onset of premenstrual syndrome in the last year, from pandemic?	
NO YES	138 (47.1%) 155 (52.9%)
Have you changed your physical activity in the last year, from pandemic?	
NO INCREASE DECREASE	72 (24.6%) 79 (27%) 142 (48.4%)

The inclusion criteria of the present study were: female students from medical school who were > 18 years old and accepted to be enrolled on this research. The exclusion criteria were: the use of contraceptive hormones, pregnancy and diagnosis of early menopause.

Informed consent was obtained from all the participants in the study. Statistical analysis was performed using the Statistical Package for Social Science (SPSS version 27.0, SPSS Inc., Chicago, IL). A Pearson chi-square test was assessed to evaluate the difference between the characteristics of the menstrual cycle and the scores obtained with the questionnaires. A *p*-value less than 0.05 was considered statistically significant.

## Results

### General profile of menstrual cycle and life habits in the population during pandemic

The data obtained showed that the incidence of menstrual alterations that began during the pandemic was 45.4%. In particular, oligomenorrhea and polymenorrhea have been observed in 24.9% and 12.6% of students, respectively. Amenorrhea has been detected in 7.8% of total cases with *p*-value > 0.05. Moreover, dysmenorrhea has been found in 12.6% of total cases, while premenstrual syndrome has been identified in 52.9% of students during the pandemic (See Table 3).

**Table 3:** Incidence of menstrual cycle’s alterations during pandemic

		Number/total %	Number/total %
Menstrual cycle’s alterations	Oligomenorrhea	73/293 (24.9%)	133/293 (45.4%)
	Polymenorrhea	37/293 (12.6%)	
	Amenorrhea	23/293 (7.8%)	
Dysmenorrhea		37/293 (12.6%)	
Premenstrual syndrome		155/293 (52.9%)	

### Menstrual alterations and traumatic impact of the event COVID-19

Statistical analysis was performed by analysing two study groups. The first group was characterised by students with an Impact of Event Scale (IES) score lower than 33 (42.3%), which means the absence of a post-traumatic stress. The second group was characterised by students with an IES score higher than 33 (57.7%), which indicates the presence of stress which was related to the pandemic. (Table 4) A positive score at IES has been detected in 63% of students with oligomenorrhea, 54% with polymenorrhea, and 73.9% with amenorrhea (Table 5). A statistically significant association has been observed between specific Impact of Event Scale test areas and menstrual disorders, showing stress symptoms in students with the onset of menstrual irregularities during the pandemic. In particular, an alteration in the interval of menstrual cycles was found in students who more easily rethink images associated with the traumatic event (47.3%) compared with those who had no rethink images (36.6%) (χ²:21.31, *p* = 0.006). Polymenorrhea was associated with an altered mood, determined by state alertness (73.2% vs. 82.7%. χ²:21.05, *p* = 0.007), a significant increase in irritability and angry (56.8% vs. 83.3%. χ²: 17.09, *p* = 0.029), a reduction in the ability to concentrate (68.8% vs. 74.5%. χ²:31.30, *p* < 0.001), and difficulty to not talking about the event (37.1% vs. 52.7%. χ²:18.66, *p* = 0.017). Instead, amenorrhea was associated with loss of interest regarding the traumatic subject (38.5% vs. 56.5%. χ²:17.09, *p* = 0.034). Finally, premenstrual syndrome was associated with a state of nervousness and fear (50% vs. 63.8%. χ²:12.99, *p* = 0.011), a greater ease in feeling irritable and angry (70.3% vs. 80.6%. χ²:11.12, *p* = 0.025) and a greater tendency to recreate images associated with the traumatic event (37.7% vs. 43.2%. χ²:16.57, *p* = 0.002). The presence of premenstrual syndrome was associated with physical symptoms, such as nausea, vomiting and increased heart rate (20.3% vs. 34.8%. χ²:16.57, *p* = 0.002) and with a sensation of reliving the same emotions (31.1% vs. 41.3%. χ²:31.91, *p* = 0.008) and a difficulty in concentrating (63.7% vs. 70.9%. χ²:12.14, *p* = 0.016). The traumatic event can have also affected the weight change. An increase or decrease in weight is also associated with a difficulty in not being upset about the event (50% vs. 66.4%. χ²:16.41, *p* = 0.037) and the attempt to eliminate the event from memory (29.5% vs. 36.6%. χ²:9.82, *p* = 0.043) with a tendency to feel irritable and angry (63.5% vs. 77%. χ²:14.95, *p* = 0.060). On the other hand, the results obtained from the Impact of Event Scale test’s total score demonstrated the absence of a statistically significant association between alteration of the menstrual cycle and the likely presence of an overt post-traumatic stress disorder. See Supplementary materials for these data.

**Table 4:** Association between menstrual cycle characteristics, clinical skills and tests’ score

	SCORE IES-R			SCORE BDI		
	<=32 (tot124)	>=33 (tot 169)	*p*	<=14 (tot 171)	>=15 (tot122)	*p*
Age (mean)	23.4	22.9	0.236	22.9	23.2	0.343
Are you sexually active?						
NO	38 (30.6%)	50 (29.6%)		52 (30.4%)	36 (29.5%)	
YES	86 (69.4%)	119 (70.4%)	0.845	119 (69.6%)	86 (70.5%)	0.487
Have you noticed any changes in your menstrual rhythm in the last year, from pandemic?						
NONE INCREASE OF INTERVAL	80 (64.5%) 27 (21.8%)	103 (60.9%) 46 (27.2%)		121 (70.8%) 32 (18.7%)	62 (50.8%) 41 (33.6%)	
DECREASE OF INTERVAL	17 (13.7%)	20 (11.8%)	0.550	18 (10.5%)	19 (15.6%)	0.02*
How often have you had your period in the last year, from pandemic?						
21–35 GG > 35	110 (88.7%) 11 (8.9%)	130 (88.8%) 18 (10.7%)		162 (94.7%) 9 (5.3%)	98 (80.3%) 20 (16.4%)	
< 21	3 (2.4%)	1 (0.6%)	0.372	0	4 (3.3%)	< 0.001*
Have you had bouts of amenorrhea in the last year, from pandemic?						
NO	118 (95.2%)	152 (89.9%)		164 (95.9%)	106 (86.9%)	
YES	6 (4.8%)	17 (10.1%)	0.101	7 (4.1%)	16 (13.1%)	0.005*
Do you suffer from dysmenorrhea?						
NO YES, IT HAS INCREASED IN THE LAST YEAR, FROM PANDEMIC YES, IT HAS DECREASED IN THE LAST YEAR, FROM PANDEMIC	72 (58.1%) 12 (9.7%) 5 (4%)	113 (66.9%) 25 (14.8%) 7 (4.1%)		116 (67.8%) 16 (9.4%) 6 (3.5%)	69 (56.6%) 21 (17.2%) 6 (4.9%)	
NO ALTERATION IN THE LAST YEAR, FROM PANDEMIC	35 (28.2%)	24 (14.2%)	0.025	33 (19.3%)	26 (21.3%)	0.145
How do you consider the amount of your menstrual cycle?						
NORMAL ABUNDANT	78 (62.9%) 33 (26.6%)	97 (57.4%) 45 (26.6%)		104 (60.8%) 47 (27.5%)	71 (59.7%) 31 (25.4%)	
RARE	13 (10.5%)	27 (16%)	0.378	20 (11.7%)	20 (16.4%)	0.510
Have you noticed any changes in the amount of your menstrual cycle in the last year. from pandemic?						
NORMAL ABUNDANT	88 (71%) 14 (11.3%)	101 (59.8%) 28 (16.6%)		123 (71.9%) 23 (13.5%)	66 (54.1%) 19 (15.6%)	
RARE	22 (17.7%)	40 (23.7%)	0.137	25 (14.6%)	37 (30.3%)	0.002*
Have you noticed any changes in your body weight in the last year, from pandemic?						
NO INCREASE	49 (39.5%) 45 (36.3%)	83 (49.1%) 41 (24.3%)		83 (48.5%) 51 (29.8%)	49 (40.2%) 35 (28.7%)	
DECREASE	30 (24.2%)	45 (26.6%)	0.076	37 (21.6%)	38 (31.1%)	0.161
Have you noticed the onset of premenstrual syndrome in the last year, from pandemic?						
NO	54 (43.5%)	84 (49.7%)		84 (49.1%)	54 (44.3%)	
YES	70 (56.5%)	85 (50.3%)	0.297	87 (50.9%)	68 (55.7%)	0.241
Have you changed your physical activity in the last year, from pandemic?						
NO INCREASE	29 (23.4%) 27 (21.8%)	43 (25.4%) 52 (30.8%)		49 (28.7%) 41 (24%)	23 (18.9%) 18 (31.1%)	
DECREASE	68 (54.8%)	74 (43.8%)	0.131	81 (47.4%)	61 (50%)	0.120

LEGEND:

BDI: Beck Depression Inventory

IES-R: Impact of Event Scale

**Table 5:** Association among menstrual changes, impact of event scale and beck depression inventory positive score

	Menstrual cycle’s alterations n/total (%)			TOT Menstrual cycle alteration n/total (%)	Dysmenorrhea n/total (%)	Premenstrual syndrome n/total (%)
	Oligomenorrhea	Polymenorrhea	Amenorrhea			
IES-R >=33	46/73 (63%)	20/37 (54%)	17/23 (73.9%)	83/133 (62.4%)	25/37 (67.5%)	85/155 (54.8%)
BDI >=15	41/73 (56.2%)	19/37 (51.3%)	16/23 (69.5%)	76/133 (57.1%)	21/37 (56.7%)	68/155 (43.8%)

### Menstrual alterations and depressive symptoms during pandemic

The Beck Depression Inventory was administered for the psychometric assessment of mood depression. A clinically significant value greater than or equal to 15 was observed in 41.7% of students, with the possible presence of depressive symptoms, while a cut-off point minor than 15 was found in 58.3% of students, indicating the likely absence of depressive symptoms [34] (see Table 4). Students who presented symptoms of depression reported a statistically significant and higher frequency of altered duration of the menstrual cycle (5.3% vs. 19.6%. χ²:16.184, *p* < 0.001), as well as for insomnia. Students who did not report symptoms of depression present less frequently menstrual cycle alteration in rhythm (70.7% vs. 50.8%. χ²:12.308, *p* = 0.002) and quantity (71.9% vs. 54.1%. χ²:12.036, *p* = 0.002), compared to the group with signs of depression. In particular, statistical analysis showed that the students who did not report sadness and sleep disturbances had less frequent changes in the menstrual cycle (χ²:26.68 *p* < 0.001; χ²:25.79 *p* < 0.001). In addition, a significant reduction (*p* < 0.001) in menstrual flow was demonstrated in students (χ²:26.83) with feeling of failure. A general alteration in the duration of the menstrual cycle was found in girls who perceived their physical aspect negatively (χ²:22.88 *p* < 0.001). A positive score at Beck Depression Inventory was found in 63% of students affected by oligomenorrhea and 54% of our population with polymenorrhea. Amenorrhea was three times more common in women with a positive score for depressive symptoms (4.1% vs. 13.1%. χ²:8.010, *p* = 0.005), and it is associated with a loss of interest in other people (χ²:7.81 *p* = 0.050). This result has been confirmed by a lower incidence of amenorrhea found in girls who do not report sadness (χ²:16.87 *p* < 0.001), who sleep well (χ²:38.21 *p* < 0.001) and who did not present problems with nutrition (χ²:16.21 *p* = 0.034) and self-disappointment (χ²:9.93 *p* = 0.019). Finally, 43.8% of students with premenstrual syndrome showed a Beck Depression Inventory score higher than 15 (Table 5). The presence of premenstrual syndrome in the last year is more common in women who report sadness, a sense of dissatisfaction and who perceived their body image negatively (χ²:12.71, *p* = 0.005; χ²:8.87, *p* = 0.031; χ²:9.89, *p* = 0.020).

### Menstrual alterations and sleep disturbances during pandemic

Students with menstrual cycle alterations and premenstrual syndrome, that started during the pandemic, presented trouble falling asleep, trouble staying asleep, early and nocturnal awakenings, and suffered from having nightmares about the pandemic (See Table 6). In particular, an alteration of menstrual cycles was more frequent in students who had insomnia with trouble staying asleep (67.2%) or with difficulties falling asleep (69.1%), compared with students who did not suffer from insomnia, trouble staying asleep (46.9%) (χ²:15.75, *p* = 0.047) or with difficulties falling asleep (45.9%) (χ²:17.17, *p* = 0.028). In addition, premenstrual syndrome was associated with trouble staying asleep (49.3% vs. 56.8%. χ²:12.81, *p* = 0.012) and difficulty in falling asleep (48.5% vs. 60%. χ²:17.10 *p* = 0.002). Furthermore, early and nocturnal awakenings were found respectively in 28.6% and 9.8% of students with menstrual alterations, such as polymenorrhea, oligomenorrhea or amenorrhea, and respectively in 16.1% and 7.7% of girls with premenstrual syndrome. Finally, it also has been found that insomnia was associated with an increase or decrease in weight (46.9% vs. 58.4%, χ²:16.47 *p* = 0.036), observed during the exposition to the event. Detailed data on the association between results from the Impact of Event Scale test and Beck Depression Inventory, alterations in the menstrual cycle and the individual conditions are available in Supplementary materials.

**Table 6:** Sleep disturbances in students with menstrual cycle’s alterations

	Menstrual cycle’s alterations n/total (%)			TOT Menstrual cycle alteration n/total (%)	Premenstrual syndrome n/total (%)
	Oligomenorrhea	Polymenorrhea	Amenorrhea		
Trouble staying asleep	46/73 (63%)	24/37 (64.8%)	18/23(78.2%)	88/133 (66.2%)	88/155 (56.7%)
Trouble falling asleep	48/73 (65.7%)	28/37 (75.6%)	17/23 (73.9%)	93/133 (69.9%)	94/155 (60.6%)
Dreams about pandemic	18/73 (24.6%)	10/37 (27%)	8/23 (34.9%)	36/133 (27.1%)	49/155 (31.6%)
Early awakenings	18/73 (24.6%)	7/37 (18.9%)	13/23 (56.5%)	38/133 (28.6%)	25/155 (16.1%)
Nocturnal awakenings	8/73 (10.9%)	2/37 (5.4%)	3/23 (13%)	13/133 (9.8%)	12/155 (7.7%)

## Discussion

The wellbeing of our doctors and other National Health System workers is very high on the political agenda at the present time, particularly regarding burnout, as it has been underlined by World Health Organization. During the pandemic, difficulties for doctors in training have been exacerbated and COVID-19 experience has led to psychological consequences on current and future population of National Health System medical workers. The present study has focused on female medical student population and its specificity. In particular, our research aims to analyse the potential correlation between alterations in the menstrual cycle and the traumatic impact of the COVID-19 pandemic. Indeed, menstrual cycle’s alterations may express hidden psychological suffering in young women. In addition, we have studied the potential correlation between the incidence of depressive symptoms and sleep disturbances in female students with menstrual cycle alterations. The outcomes of the present study, in accordance with another recent study in a group of Jordan medical students [29], endorsed positively that menstrual dysfunction is significantly and directly correlated to COVID-19 pandemic stress. The presence of stress symptoms connected to COVID-19 events has been significantly associated with the menstrual cycle’ s alteration.

The literature has demonstrated extensively how stress can affect menstrual function [35] and how the hypothalamic-pituitary axis plays a central role in the pathophysiology of menstrual changes [18, 36]. Amicucci et al. [37], in a study of 993 people, demonstrated a higher incidence of stress and sleep disorders (64.3% vs. 43.1%) in younger patients than in adulthood. A positive correlation between psychological stress, specifically due to the pandemic and menstrual alteration has already been described in adults [24, 25]. Young women with cycle disorders have been shown to have more dysfunctional attitudes, such as higher levels of control, the rigidity of ideas and more difficulty coping with daily stresses than eumenorrheic women [38–40]. However, a few research focused on the psychological consequences of a pandemic on the student population [25, 41], even though the young population was one of the most targeted by the pandemic. Only one work has studied students’ menstrual cycle and premenstrual syndrome during the pandemic [28], while another work has studied students’ dysmenorrhea and its impact on the academic performance, in April 2021 [42]. Moreover, one set of research investigated the connection between post-traumatic stress disorder and premenstrual syndrome during the pandemic in a population of high school students [43].

COVID-19 infection/illness and risk of death, together with behavioural restrictions, have conditioned the daily life, particularly affecting young people whose development and life experience have been compromised for a long time. However, medical population, including physicians and trainees, was specifically affected.

The present work reports the connection between psychological stress due to the pandemic and menstrual alterations, particularly distinguishing polymenorrhea, oligomenorrhea, amenorrhea, dysmenorrhea and premenstrual syndrome, specifically in young women who are doctors in training. Previous research has shown how university students have presented high stress and sleep disturbances during the pandemic [44, 45]. This study investigated for the first time the association between the menstrual cycle’s alterations, sleep and depression during the pandemic in a female young population of medical students. Remarkably, in this study, it has been observed that sleep disorders are strictly connected to dysmenorrhea and amenorrhea due to pandemic stress. For instance, our research identifies a close association between premenstrual syndrome with sleep disturbances and mood disorders.

With regard to mood disorders, it has been found a lower presence of menstrual changes in students who did not report symptoms of depression. This could suggest that depression is implicated with the stress due to the pandemic, thus determining menstrual irregularities. Depression has been connected with both high cortisol levels and menstrual cycle alterations [20, 21]. Several studies have analysed the incidence of depression at the time of COVID − 19 in health workers. A study of 442 physicians has shown that 64.7% had symptoms of depression, 51.6% had anxiety, and 41.2% presented stress, finding higher scores in female and young participants who were in action during the pandemic [46]. For example, a cross-sectional study, conducted with 522 female nurses, has shown how their anxiety and depression levels during the pandemic affected their menstrual patterns [47].

Stress associated with the pandemic, such as restrictions, the break and/or change of university activity, may have caused psychological consequences, depression, and sleep disorders. Interestingly, amenorrhea seems to show a specific correlation with depression, as it was three times more common in women with a positive score for depressive symptoms compared to other menstrual alterations. On the contrary, a lower incidence of amenorrhea was found in girls who did not report sadness, slept well, and did not present problems with nutrition and self-disappointment. In addition, it has been shown to be associated with a loss of interest in other people. This data on amenorrhea could suggest a connection to the particular personality profile characteristics and to hidden psychological disorders which have been observed in functional hypothalamic amenorrhea [14–17].

Stress associated with the pandemic may have determined an alteration of the menstrual cycle, even if an actual post-traumatic stress disorder has not been statistically demonstrated. This might be explained by the reduction of physical stress in our population of female medical students, who were relieved from clinical activities, especially if compared to the population of physicians who worked during the pandemic. Indeed, post-traumatic stress disorder, together with menstrual alterations, have been shown in female health workers who were active during the pandemic [46, 47]. An altered menstrual cycle in the medical students’ population is seemingly more likely due to psychological stress connected to depression and its physical consequences, as well as sleep disorders. For instance, we have observed a statistically significant connection between an altered menstrual cycle and depressive symptoms. These data lead to consideration of the rising need of psychological interventions after pandemic, in medical students, with particular attention to women, which should be taken into consideration by university institutions and governments.

### Limitations of the study

No data before the pandemic has been collected, as the present study was designed after the beginning of the COVID-19 pandemic. Data from the period before the pandemic has not been not analysed. However, the questionnaire and tests were focused on students’ personal situation which began during the pandemic period, and students were invited to describe only characteristics and symptoms which started after the pandemic.

## Conclusions

Stress, menstrual alterations, mood disorders and sleep disturbances have been found in medical students during the pandemic. Nevertheless, an absence of a statistically significant association between the alteration of the menstrual cycle and the presence of post-traumatic stress has been found. Post-traumatic stress seems to have targeted people who have presented high physical stress, such as working doctors, more than doctors in training. Future studies are essential to understand the mechanisms underlying these phenomena, specifically in the female young population. Specific strategies of psychological interventions should be necessary to help medical students, in order to face not only the usual daily stress but also the consequence due to pandemic.

### Electronic supplementary material

Below is the link to the electronic supplementary material.

Supplementary Material 1

## Data Availability

The datasets used and/or analysed during the current study are available from the corresponding author on reasonable request.
