# Supplementary material for: The impact of COVID-19 on menstrual cycle’s alterations, in relation to depression and sleep disturbances: a prospective observational study in a population of medical students
Source: BMC Womens Health. 2024 Feb 19;24:130. doi: 10.1186/s12905-024-02971-x (PMC10877769; doi:10.1186/s12905-024-02971-x)
Supplement: Supplementary file 1 — Supplementary Material 1 [file 12905_2024_2971_MOESM1_ESM.pdf]

IES-R TEST Supplementary

Table S1. relationship between the menstrual cycle and the impact of the pandemic as a traumatic event. A,B: relationship with the altered duration of the menstrual cycle; C: Amenorrhea; D: Change Weight; E: Premenstrual syndrome

A **IN THE LAST YEAR ALTERATION OF MENSTRUAL CYCLE**

PATIENTS n.293 (100%)	No (183 / 62.5%)	Increase of interval (73/24.9%)	Reduction of interval (37/12.6%)	P
1.Any reminder brought back feelings about it				
- none	15 (8.2%)	10 (13.7%)	0	
-not much	47 (25.7%)	14 (19.2%)	12 (32.4%)	
-moderately	69 (37.7%)	15 (15.8 %)	11(29.7%)	
-enough	44 (24%)	25 (30.9%)	12(32.4%)	
-extremely	8 (4.4%)	9(12.3%)	2 (5.4%)	0.012*
2.I had trouble staying asleep				
. - none	58 (31.7%)	19 (26%)	5(13.5%)	
-not much	39 (21.3%)	8 (11%)	8 (21.6%)	
-moderately	30 (16.4%)	11 (15.1 %)	9 (24.3%)	
-enough	41 (22.4%)	20 (27.4%)	10(27%)	
-extremely	15 (8.2%)	15(20.5%)	5 (13.5%)	0.047*
3.Other things kept making me think about it.				
- none	25 (13.7%)	5 (6.8%)	2(5.4%)	
-not much	48 (26.2%)	18 (24.7%)	7 (18.9%)	
-moderately	66 (36.1%)	22 (30.1 %)	15 (40.5%)	
-enough	34 (18.6%)	21 (28.8%)	11 (29.7%)	
-extremely	10 (5.5%)	7(9.6%)	2 (5.4%)	0.283
4.I felt irritable and angry				
- none	10(5.5%)	2(2.7%)	1 (2.7%)	
-not much	43(23.5%)	12(16.4%)	3(8.1%)	
-moderately	51 (27.9%)	13(17.8%)	9(24.3%)	
-enough	54 (29.5%)	27(37%)	19(51.4%)	
-extremely	25 (13.7%)	19 (26%)	5 (13.3%)	0.029*
5.I avoided letting myself get upset when I thought about it or was reminded of it.				
- none	17(9.3%)	9(12.3%)	2(5.4%)	
-not much	62(33.9%)	15(20.5%)	15(40.5%)	
-moderately	51(27.9%)	27(37%)	14(37.8%)	
-enough	48(26.2%)	19(26%)	6(16.2%)	
-extremely	5(2.7%)	3(4.1%)	0	0.258
6.I thought about it when I didn't mean to				
- none	20(10.9%)	8(11%)	2(5.4%)	
-not much	52(28.4%)	18(24.7%)	8(21.6%)	
-moderately	62(33.9%)	15(20.5%)	15(40.5%)	
-enough	44(24%)	22(30.1%)	6(16.2%)	
-extremely	5(2.7%)	10(13.7%)	6(16.2%)	0.006*
7.I felt as if it hadn't happened or wasn't real.				
- none	72(39.3%)	26(35.6%)	9(24.3%)	
-not much	39(21.3%)	14(19.2%)	11(29.7%)	
-moderately	31(16.9%)	12(16.4%)	11(29.7%)	
-enough	31(16.9%)	19(26%)	5(13.5%)	
-extremely	10(5.5%)	2(2.7%)	1(2.7%)	0.247
8.I stayed away from reminders of it.				
- none	58(31.7%)	14(19.2%)	8(21.6%)	
-not much	69(37.7%)	25(34.2%)	13(35.1%)	
-moderately	35(19.1%)	22(30.1%)	10(27%)	
-enough	14(7.7%)	8(11%)	5(15.3%)	
-extremely	7(3.8%)	4(5.5%)	1(2.7%)	0.360
9.Pictures about it popped into my mind.				
. - none	59 (32.2%)	21 (28.8%)	8(21.6%)	
-not much	57 (31.1%)	12 (16.4%)	17 (45.9%)	
-moderately	46 (25.1 %)	20 (27.4 %)	4(10.8%)	
-enough	16 (8.7%)	14 (19.2%)	5 (13.5%)	
-extremely	5(2.7%)	6(8.2%)	3 (8.1%)	0.006*

10.I was jumpy and easily startled.				
. - none	30 (16.4%)	8 (11%)	6 (16.2.%)	
-not much	58 (31.7%)	15 (20.5%)	8(21.6%)	
-moderately	36 (19.7 %)	18 (24.7 %)	14(37.8%)	
-enough	41 (22.4%)	21 (28.8%)	6 (16.2.%)	
-extremely	18(9.8%)	11(15.1%)	3 (8.1%)	0.158
11.I tried not to think about it.	18(9.8%)	4 (5.5%)	2 (5.4%)	
- none	40 (21.9%)	17 (23.3 %)	11(29.7%)	
-not much	55 (30.1%)	16 (21.9%)	13(35.1%)	
-moderately	53 (29 %)	28(38.4%)	10 (27.1%)	
-enough	17 (9.3%)	8 (11%)	1 (2.7%)	
-extremely				0.439
12. I was aware that I still had a lot of feelings about it, but I didn't deal with them.				
- none	53(29%)	17 (23.3 %)	11(29.7%)	
-not much	54 (29.5%)	10 (13.7%)	8(21.6%)	
-moderately	42 (23%)	24 (32.9%)	11(29.7%)	
-enough	29 (15.8 %)	19(26%)	6(16.2%)	
-extremely	5 (2.7%)	3 (4.1%)	1 (2.7%)	0.167
13.My feelings about it were kind of numb.				
. - none	74 (40.4%)	20 (27.4%)	12 (32.4%)	
-not much	40 (21.9%)	20 (27.4%)	10(27%)	
-moderately	39(21.3%)	13 (17.8%)	10(27%)	
-enough	24 (13.1%)	16 (21.9%)	4(10.8%)	
-extremely	6(3.3%)	4 (5.5%)	1(2.7%)	0.391
14.I found myself acting or feeling like I was back at that time.				
- none	66 (36.1%)	23 (31.5%)	7 (18.9%)	
-not much	57 (31.1%)	19 (26%)	14(37.8%)	
-moderately	30 (16.4%)	13 (17.8%)	9(24.3%)	
-enough	24 (13.1%)	17 (23.3%)	5(13.5%)	
-extremely	6 (3.3%)	1(1.4%)	2(5.4%)	0.266
15.I had trouble falling asleep.				
- none	58 (31.7%)	15(20.5%)	4 (10.8%)	
-not much	41 (22.4%)	10 (13.7%)	5 (13.5%)	
-moderately	20 (10.9%)	11 (15.1%)	8 (21.6%)	
-enough	37 (20.2%)	19 (26%)	11 (29.7%)	
-extremely	27 (14.8%)	18 (24.7%)	9(24.3%)	0.028*
16.I had waves of strong feelings about it.				
- none	37 (20.2%)	8 (11%)	5 (13.5%)	
-not much	42 (23.0%)	20 (27.4%)	13 (35.1%)	
-moderately	49 (26.8%)	16 (21.9%)	8 (21.6%)	
-enough	39 (21.3%)	18 (24.7%)	7 (18.9%)	
-extremely	16 (8.7%)	11 (15.1%)	4 (10.8%)	0.440
17.I tried to remove it from my memory.				
- none	84 (45.9%)	23(31.5%)	15 (40.5%)	
-not much	46 (25.1%)	15(20.5%)	12(32.4%)	
-moderately	24 (13.1%)	13(17.8%)	6(16.2%)	
-enough	20 (10.9%)	16(21.9%)	4(10.8%)	
-extremely	9 (4.9%)	6(8.2%)	0	0.112
18.I had trouble concentrating.				
. - none	18(9.8%)	4(5.5%)	2(5.4%)	
-not much	39(21.3%)	11(15.1%)	11(29.7%)	
-moderately	37(20.2%)	6(8.2%)	7(18.9%)	
-enough	61(33.3%)	27(37%)	2(5.4%)	
-extremely	28(15.3%)	25(34.2%)	15(40.5%)	<0.001*
19.Reminders of it caused me to have physical reactions, such as sweating, trouble breathing, nausea, or a pounding heart.				
- none	95 (51.9%)	95 (51.9%)	16 (43.2%)	
-not much	39 (21.3%)	39 (21.3%)	10 (27%)	
-moderately	23 (12.6%)	23 (12.6%)	2 (5.4%)	
-enough	22 (12%)	22 (12%)	6 (16.2%)	
-extremely	4 (2.2%)	4 (2.2%)	3 (8.1%)	0.036*
20.I had dreams about it.				
- none	94(51.4%)	32(43.8%)	16(43.2%)	
-not much	41(22.4%)	17(23.3%)	11(29.7%)	
-moderately	25(13.7%)	10(13.7%)	2(5.4%)	
-enough	17(9.3%)	6(8.2%)	6(16.2%)	
-extremely	6(3.3%)	8(11%)	2(5.4%)	0.219

21. I felt watchful and on-guard.				
. - none	22(12%)	8(11%)	1(2.7%)	
-not much	27(14.8%)	8(11%)	2(5.4%)	
-moderately	41(22.4%)	18(24.7%)	17(45.9%)	
-enough	70(38.3%)	19(26%)	12(32.4%)	
-extremely	23(12.6%)	20(27.4%)	5(13.5%)	0.007*
22. I tried not to talk about it. them.				
- none	71 (38.8%)	19 (26%)	7 (18.9%)	
-not much	44 (24%)	11 (15.1%)	15 (40.5%)	
-moderately	37 (20.2%)	24 (32.9%)	7 (18.9%)	
-enough	20 (10.9%)	14 (19.2%)	6 (16.2%)	
-extremely	11 (6%)	5 (6.8%)	2 (5.4%)	0.017*

B	IN THE LAST YEAR HOW OFTEN ALTERATION OF MENSTRUAL CYCLE			
**PATIENTS n.293 (100%)**	**21-35 D (260 / 88.7%)**	**>35 D (29/9.9%)**	**<21D (4/1.4%)**	**P**
1.Any reminder brought back feelings about it				
- none	17 (65 %)	8 (27.6%)	0	
-not much	68 (26.2 %)	2 (6.9%)	3 (75%)	
moderately	88 (33.8%)	7 (24.1%)	0	
-enough	72 (27.7%)	8 (27.6%)	1 (25%)	
-extremely	15 (5.8%)	4(13.8%)	0	<0.001*
4.I felt irritable and angry				
- none	13(5%)	0	0	
-not much	49(18.8%)	9(16.4%)	0	
moderately	68(26.2%)	2(17.8%)	3(75%)	
-enough	89(34.2%)	10(37%)	1(25%)	
-extremely	41(15.8%)	8 (26%)	0	0.053
5.I avoided letting myself get upset when I thought about it or was reminded of it.				
- none	22(8.5%)	6(20.7%)	0	
-not much	85(32.7%)	5(17.2%)	2(50%)	
moderately	81(31.2%)	10(34.5%)	1(25%)	
-enough	67(25.8%)	6(20.7%)	0	
-extremely	5(1.9%)	2(6.9%)	1(25%)	0.021*
18.I had trouble concentrating.				
. - none	22(8.5%)	2(6.9%)	0	
-not much	57(21.9%)	1(3.4%)	3(75%)	
moderately	48(18.5%)	2(6.9%)	0	
-enough	79(30.4%)	11(37.9%)	0	
-extremely	54(20.8%)	13(44.8%)	1(25%)	<0.001*
19.Reminders of it caused me to have physical reactions, such as sweating, trouble breathing, nausea, or a pounding heart.				
- none	132 (50.8%)	14 (48.3%)	1 (25%)	
-not much	60 (23.1%)	4 (13.8%)	0	
moderately	26 (10%)	2 (6.9%)	0	
-enough	31 (11.9%)	4 (13.8%)	3 (75%)	
-extremely	11 (4.2%)	5 (17.2%)	0	0.003*

C **IN THE LAST YEAR AMENORRHEA?**

PATIENTS n.293 (100%)		NO 270 (92.2%)	YES 23 (7.8%)	P
1.Any reminder brought back feelings about it				
	- none	20(7.4%)	5(21.7%)	
	-not much	73(27%)	0	
	-moderately	86(31.9%)	9(39.1%)	
	-enough	72(26.7%)	9(39.1%)	
	-extremely	19(7%)	0	0.006*
2.I had trouble staying asleep .				
	- none	80 (29.6%)	2(8.7%)	
	-not much	52 (19.3%)	3(13%)	
	-moderately	46 (17%)	4(17.4 %)	
	-enough	61 (22.6%)	10(43.5%)	
	-extremely	31 (11.5%)	4(17.4%)	0.085
3.Other things kept making me think about it.				
	- none	31 (11.5%)	1(4.3%)	
	-not much	68 (25.2%)	4(17.4 %)	
	-moderately	92 (34.1%)	2(8.7%)	
	-enough	60 (22.2%)	12(52.2%)	
	-extremely	19 (7%)	4(17.4%)	0.408
4.I felt irritable and angry				
	- none	12(4.4%)	1 (4.3%)	
	-not much	54(20%)	12(16.4%)	
	-moderately	71(26.3%)	13(17.8%)	
	-enough	88(32.8%)	27(37%)	
	-extremely	45 (16.7%)	19 (26%)	0.278
5.I avoided letting myself get upset when I thought about it or was reminded of it.				
	- none	26(9.6%)	2(8.7%)	
	-not much	86(31.9%)	6(26.1%)	
	-moderately	82(30.4%)	10(43.5%)	
	-enough	70(25.9%)	13(4.1%)	
	-extremely	6(2.2%)	2(8.7%)	0.194
6.I thought about it when I didn't mean to				
	- none	28(10.4%)	2(8.7%)	
	-not much	73(27%)	5(21.7%)	
	-moderately	84(31.1%)	8(34.8%)	
	-enough	66(24.4%)	6(26.1%)	
	-extremely	19(7%)	2(8.7%)	0.976
7.I felt as if it hadn't happened or wasn't real.				
	- none	101(37.4%)	6(26.1%)	
	-not much	60(22.2%)	4(17.4%)	
	-moderately	50(18.5%)	4(17.4%)	
	-enough	47(17.4%)	8(34.8%)	
	-extremely	12(4.4%)	1(4.3%)	0.355
8.I stayed away from reminders of it.				
	- none	75(27.8%)	5(21.7%)	
	-not much	98(36.3%)	9(39.1%)	
	-moderately	63(23.3%)	4(17.4%)	
	-enough	23(8.5%)	4(17.4%)	
	-extremely	11(4.1%)	1(4.3%)	0.650
9.Pictures about it popped into my mind. .				
	- none	82(30.4%)	6(26.1%)	
	-not much	79(29.3%)	7(30.4%)	
	-moderately	63(23.3%)	7(30.4 %)	
	-enough	32 (11.9%)	3(13%)	
	-extremely	14(5.2%)	0	0.772
10.I was jumpy and easily startled. .				
	- none	42 (15.6%)	2(8.7%)	
	-not much	75 (27.8%)	6(26.1%)	
	-moderately	62 (23%)	6(26.1%)	
	-enough	61(22.6%)	7(30.7%)	
	-extremely	30(11.1%)	2(8.7%)	0.835

11.I tried not to think about it.<br>- none<br>-not much<br>-moderately<br>-enough<br>-extremely	22(8.1%)<br>60(23%)<br>79(29.3%)<br>82 (30.4%)<br>25(9.3%)	2(8.7%)<br>6(26.1%)<br>5 (21.7%)<br>9(39.1%)<br>1 (4.3%)	0.806
12. I was aware that I still had a lot of feelings about it, but I didn't deal with them.<br>- none<br>-not much<br>-moderately<br>-enough<br>-extremely	76(28.1%)<br>68(25.2%)<br>71(26.3%)<br>47(26.3%)<br>8(3%)	5(21.7%)<br>4(17.4%)<br>6(26.1%)<br>7(30.4%)<br>1 (4.3%)	0.571
13.My feelings about it were kind of numb.<br>. -<br>- none<br>-not much<br>-moderately<br>-enough<br>-extremely	101 (37.4%)<br>65 (24.1%)<br>59(21.9%)<br>36(13.3%)<br>9(3.3%)	5(21.7%)<br>5(21.7%)<br>3(13%)<br>8 (34.8%)<br>2 (8.7%)	0.034*
14.I found myself acting or feeling like I was back at that time.<br>- none<br>-not much<br>-moderately<br>-enough<br>-extremely	89 (33%)<br>88 (32.6%)<br>44 (16.3%)<br>40 (14.8%)<br>9 (3.3%)	7 (30.4%)<br>2 (8.7%)<br>8 (34.8%)<br>6 (26.1%)<br>0	0.032*
15.I had trouble falling asleep.<br>- none<br>-not much<br>-moderately<br>-enough<br>-extremely	74 (27.4%)<br>53 (19.6%)<br>37 (13.7%)<br>58 (21.5%)<br>48 (17.8%)	3(13%)<br>3(13%)<br>2 (8.7%)<br>9 (39.1%)<br>6 (26.1%)	0.183
16.I had waves of strong feelings about it.<br>- none<br>-not much<br>-moderately<br>-enough<br>-extremely	45 (16.7%)<br>73 (27%)<br>67 (24.8%)<br>57 (21.1%)<br>28 (10.4%)	5(21.7%)<br>2 (8.7%)<br>6 (26.1%)<br>7 (30.4%)<br>3(13%)	0.392
17.I tried to remove it from my memory.<br>- none<br>-not much<br>-moderately<br>-enough<br>-extremely	116 (43%)<br>66 (24.4%)<br>38 (14.1%)<br>36 (13.3%)<br>14 (5.2%)	6 (26.1%)<br>7 (30.4%)<br>5(21.7%)<br>4(17.4%)<br>1 (4.3%)	0.577
18.I had trouble concentrating.<br>. - none<br>-not much<br>-moderately<br>-enough<br>-extremely	21(7.8%)<br>59(21.9%)<br>48(17.8%)<br>81(30%)<br>61(22.6%)	3(13%)<br>2 (8.7%)<br>2 (8.7%)<br>9 (39.1%)<br>7 (30.4%)	0.326
19.Reminders of it caused me to have physical reactions, such as sweating, trouble breathing, nausea, or a pounding heart.<br>- none<br>-not much<br>-moderately<br>-enough<br>-extremely	136 (50.4%)<br>59 (21.9%)<br>28 (10.4%)<br>33 (12.2%)<br>14 (5.2%)	11 (47.8%)<br>5 (21.7%)<br>0<br>5 (21.7%)<br>2 (8.7%)	0.358
20.I had dreams about it.<br>- none<br>-not much<br>-moderately<br>-enough<br>-extremely	129(47.8%)<br>67(24.8%)<br>33(12.2%)<br>10(27%)<br>14(5.2%)	13(56.6%)<br>2 (8.7%)<br>4(17.4%)<br>2 (8.7%)<br>2 (8.7%)	0.458
21. I felt watchful and on-guard. .<br>. - none<br>-not much<br>-moderately<br>-enough<br>-extremely	29(10.7%)<br>35(13%)<br>71(26.7%)<br>91(33.7%)<br>43(15.9%)	2 (8.7%)<br>2 (8.7%)<br>4(17.4%)<br>10 (43.5%)<br>5 (21.7%)	0.714
22. I tried not to talk about it. them.<br>- none<br>-not much<br>-moderately<br>-enough<br>-extremely	92 (34.1%)<br>65 (24.1%)<br>58 (21.5%)<br>38 (14.1%)<br>17 (6.3%)	5 (21.7%)<br>5(21.7%)<br>10 (43.5%)<br>2 (8.7%)<br>1 (4.3%)	0.198

D

AMENORRHEA

**PATIENTS n.293 (100%)**	**No (132 / 45.1%)**	**Increse (86/29.4%)**	**Reduction (75/25.6%)**	**P**
1.Any reminder brought back feelings about it				
- -none	13 (8.2%)	3 (3.5%)	9 (12%)	
-not much	33 (25.7%)	22 (25.6%)	18 (24%)	
-moderately	38 (37.7%)	34 (39.5 %)	23 (30.7%)	
-enough	41 (24%)	21 (24.4%)	19 (25.3%)	
-extremely	7 (4.4%)	6(7%)	6 (8%)	0.485
2.I had trouble staying asleep				
- -none	46 (34.8%)	20 (23.3%)	16(21.3%)	
-not much	24 (18.2%)	18 (20.9%)	13 (17.3%)	
-moderately	22 (16.7%)	16 (18.6 %)	12 (16%)	
-enough	27 (20.5%)	16 (18.6 %)	28 (37.3%)	
-extremely	13 (9.8%)	16 (18.6 %)	6 (8%)	0.036*
3.Other things kept making me think about it.				
- none	14 (10.6%)	11 (12.8%)	7(9.3%)	
-not much	33 (25%)	25 (29.1 %)	15 (20%)	
-moderately	50 (37.9%)	25 (29.1 %)	28 (37.3%)	
-enough	24 (18.2%)	22(25.6 %)	20 (26.7%)	
-extremely	11 (8.3%)	3 (3.5 %)	5 (6.7%)	0.537
4.I felt irritable and angry				
- none	8(61.1%)	4(4.7%)	1(1.3%)	
-not much	27 (20.5%)	14(16.3%)	17(22.7%)	
-moderately	38(28.8%)	20(23.3%)	15(20%)	
-enough	32(24.2%)	34(39.5%)	34(45.3%)	
-extremely	27 (20.5%)	14(16.3%)	8(10.7%)	0.060
5.I avoided letting myself get upset when I thought about it or was reminded of it.				
- none	15(11.4%)	7(8.1%)	6(8%)	
-not much	51(38.6%)	20(23.3%)	21(28%)	
-moderately	37 (28%)	35(40.7%)	20(26.7%)	
-enough	27 (20.5%)	23(26.7%)	23(30.7%)	
-extremely	2 (1.5%)	1(1.2%)	5(6.7%)	0.037*
6.I thought about it when I didn't mean t				
- none	9 (6.8%)	11(12.8%)	10(13.3%)	
-not much	33 (25%)	27(31.4%)	18(24%)	
-moderately	45 (34.1%)	22(25.6%)	25(33.3%)	
-enough	34 (25.8%)	22(25.6%)	16(21.3%)	
-extremely	11 (8.3%)	4(4.7%)	6(8%)	0.575
7.I felt as if it hadn't happened or wasn't real.				
- none	56(42.4%)	26 (30.2%)	25 (33%)	
-not much	25(18.9%)	23 (26.7%)	16 (21.3%)	
-moderately	22(16.7%)	19 (22%)	13 (17.3%)	
-enough	23 (17.4%)	14 (16.3%)	18 (24%)	
-extremely	6 (4.5%)	4 (4.7%)	3 (4%)	0.612
8.I stayed away from reminders of it.				
- none	42 (31.8%)	23 (26.7%)	15 (20%)	
-not much	47 (35.6%)	35 (40.7%)	25 (33.3%)	
-moderately	30 (22.7%)	21 (24.4%)	16 (21.3%)	
-enough	8 (6.1%)	5 (5.8%)	14 (18.7%)	
-extremely	5 (3.8%)	2 (2.3%)	5 (6.7%)	0.060
9.Pictures about it popped into my mind.				
. - none	41 (31.1%)	26 (30.2%)	21 (28%)	
-not much	35 (26.5 %)	23 (26.7%)	28 (37.3%)	
-moderately	36 (27.3%)	21 (24.4 %)	13 (17%)	
-enough	12 (9.1%)	12 (14%)	11 (14.7%)	
-extremely	8 (6.1%)	4(4.7%)	2 (2.7%)	0.521
10.I was jumpy and easily startled.				
. - none	23 (17.4%)	11 (12%)	10 (13%)	
-not much	35 (26%)	27 (31.4%)	19 (25.3%)	
-moderately	27 (20.5 %)	18 (20.9 %)	23 (30.7%)	
-enough	30 (22%)	22 (25.6%)	16 (12%)	
-extremely	17 (12%)	8 (9.3%)	7 (9.3%)	0.738

11.I tried not to think about it.				
- none	10 (7.6%)	9 (10.5%)	5 (6.7%)	
-not much	37 (28%)	20 (23.3 %)	11 (14.7%)	
-moderately	41 (31.1%)	19 (22.1%)	24 (32%)	
-enough	32 (24.2 %)	31 (36%)	28 (37.3%)	
-extremely	12 (9.1%)	7 (8.1%)	7 (9.3%)	0.262
12. I was aware that I still had a lot of feelings about it, but I didn't deal with them.				
- none	34 (25.8%)	24 (27.9 %)	23 (30.7%)	
-not much	36 (27.3%)	20 (23.3%)	16 (12.3%)	
-moderately	33 (25%)	22 (25.6%)	22 (29.3%)	
-enough	26 (16.7 %)	15 (17.4%)	13 (17v3%)	
-extremely	3 (2.3%)	5 (5.8%)	1 (1.3%)	0.770
13.My feelings about it were kind of numb.				
. - none	53 (40.2%)	30 (34%)	23 (30.7%)	
-not much	27 (20.5%)	23 (26%)	20 (26.7%)	
-moderately	33 (25%)	15 (17%)	14 (18.7%)	
-enough	16 (12.1%)	14 (16%)	14 (18%)	
-extremely	3(2.3%)	4 (4.7%)	4(5.3%)	0.528
14.I found myself acting or feeling like I was back at that time.				
- none	45 (34.1%)	27(31.4%)	24 (32%)	
-not much	38 (28.4%)	26 (30.2%)	26 (34.7%)	
-moderately	25 (18.9%)	15 (17.4%)	12 (16%)	
-enough	23 (17.4%)	13 (15.1%)	10 (13.3%)	
-extremely	1(0.8%)	5(5.8%)	3 (4%)	0.639
15.I had trouble falling asleep.				
- none	43 (32.6%)	16 (18%)	18 (24%)	
-not much	23 (17.4%)	19 (22.1%)	14 (18.7%)	
-moderately	16 (12.1%)	14 (16.3%)	9 (12%)	
-enough	27 (20.5%)	19 (22.1%)	21 (28%)	
-extremely	23 (17.4%)	18 (20.9%)	13 (17.3%)	0.524
16.I had waves of strong feelings about it				
- none	22 (16.7%)	12 (14%)	16 (21.3%)	
-not much	35 (26.5%)	22 (25.6%)	18 (24%)	
-moderately	35 (26.5%)	19 (22.1%)	19 (25.3%)	
-enough	26 (19.7%)	23 (26.7%)	15 (20%)	
-extremely	14 (10.6%)	10 (11.6%)	7(9.3%)	0.909
17.I tried to remove it from my memory.				
- none	58 (43%)	32 (37.2%)	32 (42.7%)	
-not much	35 (26.5%)	23 (26.7%)	15 (20%)	
-moderately	18 (13.6%)	16 (18.6%)	9(12%)	
-enough	10 (7.6%)	13 (15.1%)	17 (22.7%)	
-extremely	11 (8.3%)	2 (2.3%)	2 (2.7%)	0.043*
18.I had trouble concentrating.				
. - none	16 (12.1%)	3(3.5%)	5 (6.7%)	
-not much	29 (22%)	15 (17.4%)	17 (22.7%)	
-moderately	24 (18.2%)	16(18.6%)	10 (13.3%)	
-enough	36 (27.3%)	31 (36%)	23 (30.7%)	
-extremely	27 (20.5%)	21 (24.4%)	20 (26.7%)	0.368
19.Reminders of it caused me to have physical reactions, such as sweating, trouble breathing, nausea, or a pounding heart.				
- none	64 (48.5%)	46 (53.5%)	37 (49.3%)	
-not much	28 (21%)	21 (24.4%)	15 (20%)	
-moderately	19 (14%)	4 (4.7%)	5 (6%)	
-enough	14 (10.6%)	12 (14%)	12 (16%)	
-extremely	7 (5.3%)	3 (3.5%)	6 (8%)	0.310
20.I had dreams about it.				
- none	61 (46.2%)	41 (47.7%)	40 (53.3%)	
-not much	29 (22%)	24 (27.9%)	16 (12.3%)	
-moderately	22 (16.7%)	11 (12.8%)	4 (5.3%)	
-enough	11 (8.3%)	9 (10.5%)	(12%)	
-extremely	9 (6.8%)	1(1.2%)	6 (8%)	0.185
21. I felt watchful and on-guard. .				
. - none	15 (11.4%)	11 (12.8%)	5 (6.7%)	
-not much	18 (13%)	11 (12.8%)	8 (10.7%)	
-moderately	38 (28.8%)	15 (17.4%)	23 (30.7%)	
-enough	41 (31.1%)	34 (39.5%)	26 (34.7%)	
-extremely	20 (15.2%)	15 (17.4%)	13 (17.3%)	0.574
22. I tried not to talk about it. them.				
- none	49 (37.1%)	27 (31.4%)	21 (28%)	
-not much	30 (22.7%)	28 (38.6%)	12 (16%)	
-moderately	29 (22%)	19 (22.1%)	20 (26.7%)	
-enough	15 (11%)	10 (11.6%)	15 (20%)	
-extremely	9 (6.8%)	2 (2.3%)	7 (9.3%)	0.115

PATIENTS n.293 (100%)	NO (138 / 47.1%)	YES (155/52.9%)	P
1.Any reminder brought back feelings about it			
- none	16 (11%)	9(5.8%)	
-not much	35 (25.4%)	38 (24.5%)	
-moderately	48 (34.8%)	47 (30.3%)	
-enough	34 (24.6%)	47 (30.3%)	
-extremely	5(3.6%)	14 (9%)	0.112
2.I had trouble staying asleep			
- none	47 (34.1%)	35 (22.6%)	
-not much	23 (16.7%)	32 (20.6%)	
-moderately	26 (18.8%)	24 (15.5%)	
-enough	34 (24.6%)	37 (23.9%)	
-extremely	8 (5.8%)	27 (17.4%)	0.012*
3.Other things kept making me think about it.			
- none	16 (11.6%)	16 (10.3%)	
-not much	29 (21%)	44 (28.4%)	
-moderately	55 (39.9%)	48 (31%)	
-enough	32 (23.2%)	34 (21%)	
-extremely	6 (4.3%)	13 (8.4%)	0.265
4.I felt irritable and angry			
- none	10 (7.2%)	3 (1.9%)	
-not much	31 (22.5%)	27 (17.4%)	
-moderately	36 (26.1%)	37 (23.9%)	
-enough	46 (33.3%)	54 (34.8%)	
-extremely	15 (10.9%)	34 (21.9%)	0.025*
5.I avoided letting myself get upset when I thought about it or was reminded of it.			
- none	14 (10.1%)	14 (9%)	
-not much	43 (31.2%)	49 (31.6%)	
-moderately	44 (31.9%)	48 (31%)	
-enough	33 (23.9%)	40 (25.8%)	
-extremely	4 (2.9%)	4(2.6%)	0.993
6.I thought about it when I didn't mean to			
- none	14 (10.1%)	16 (10.3%)	
-not much	41 (29.7%)	37 (23.9%)	
-moderately	49 (35.5%)	43 (27.7%)	
-enough	30 (21.7%)	42 (27.1%)	
-extremely	4(2.9%)	17 (11%)	0.043*
7.I felt as if it hadn't happened or wasn't real.			
- none	51 (37%)	56 (36.1%)	
-not much	29 (21%)	35 (22.6%)	
-moderately	21 (15.2%)	33 (21.3%)	
-enough	31 (22.5%)	24 (15.5%)	
-extremely	6 (4.3%)	7 (4.5%)	0.485
8.I stayed away from reminders of it.			
- none	45 (32.6%)	35 (22.6%)	
-not much	43 (31.2%)	64 (41.3%)	
-moderately	29 (21%)	38 (24.5%)	
-enough	18 (13%)	9(5.8%)	
-extremely	3(2.2%)	9 (5.8%)	0.020*
9.Pictures about it popped into my mind.			
. - none	43 (31.2%)	45 (29%)	
-not much	43 (31.2%)	43 (27.7%)	
-moderately	41 (29.7%)	29 (18.7%)	
-enough	9 (6.5%)	26 (16.8%)	
-extremely	2 (1.4%)	12 (7.7%)	0.02*
10.I was jumpy and easily startled.			
. - none	29 (21%)	15 (9.7%)	
-not much	40 (29%)	41 (26.5%)	
-moderately	31 (22.5%)	37 (23.9%)	
-enough	30 (21.7%)	38 (24.5%)	
-extremely	8 (5.8%)	24 (15.5%)	0.011*
11.I tried not to think about it.			
- none	10 (7.2%)	14 (9%)	
-not much	41 (29.7%)	27 (17.4%)	
-moderately	33 (23.9%)	51 (32.9%)	
-enough	44 (31.9%)	47 (39.3%)	
-extremely	10 (7.2%)	16 (10.3%)	0.094*

12. I was aware that I still had a lot of feelings about it, but I didn't deal with them.			
- none	48 (34.8%)	33 (21.3%)	
-not much	28 (20.3%)	44 (28.4%)	
-moderately	40 (29%)	37 (23.9%)	
-enough	22 (17.9%)	32 (20.6%)	
-extremely	0	9(5.8%)	0.003*
13.My feelings about it were kind of numb. .			
- none	52(37.7%)	54 (34.8%)	
-not much	35 (25.4%)	35 (26.6%)	
-moderately	31 (22.5%)	31 (20%)	
-enough	15 (10.9%)	29 (18.7%)	
-extremely	5 (3.6%)	6 (3.9%)	0.462
14.I found myself acting or feeling like I was back at that time.			
- none	56 (40.6%)	40 (25.8%)	
-not much	39 (28.3%)	51 (32.9%)	
-moderately	22 (15.9%)	30 (19.4%)	
-enough	21 (15.2%)	25 (16.1%)	
-extremely	0	9(5.8%)	0.008*
15.I had trouble falling asleep.			
- none	42 (30.4%)	35 (22.6%)	
-not much	29 (21%)	27 (17.4%)	
-moderately	22 (15.9%)	17 (11%)	
-enough	33 (23.9%)	34 (21.9%)	
-extremely	12 (8.7%)	42 (27.1%)	0.002*
16.I had waves of strong feelings about it.			
- none	34 (24.6%)	16 (10.3%)	
-not much	27 (19.6%)	48 (31%)	
-moderately	43 (31.2%)	30 (19.4%)	
-enough	25 (18.1%)	39 (25.2%)	
-extremely	9 (6.5%)	22 (14.2%)	<0.001*
17.I tried to remove it from my memory.			
- none	59 (42.8%)	63 (40.6%)	
-not much	39 (28.3%)	34 (21.9%)	
-moderately	18 (13%)	25 (16.1%)	
-enough	17 (12.3%)	23 (14.8%)	
-extremely	5 (3.6%)	10 (6.5%)	0.524
18.I had trouble concentrating.			
. - none	18 (13%)	Sei (3.9%)	
-not much	32 (23.2%)	29 (18%)	
-moderately	25 (18.1%)	25 (16.1%)	
-enough	38 (27%)	52 (33.5%)	
-extremely	25 (18.1%)	43 (27.7%)	0.016*
19.Reminders of it caused me to have physical reactions, such as sweating, trouble breathing, nausea, or a pounding heart.			
- none	82 (59.4%)	65 (41.9%)	
-not much	28 (20.3%)	36 (23.2%)	
-moderately	13 (9.4%)	15 (9.7%)	
-enough	14 (10.1%)	24 (15.5%)	
-extremely	1(0.7%)	15 (9.7%)	0.002*
20.I had dreams about it.			
- none	76 (55.1%)	66 (42.6%)	
-not much	29 (21%)	40 (25.8%)	
-moderately	15 (10.9%)	22 (14.2%)	
-enough	13 (9.4%)	16 (10.3%)	
-extremely	5 (3.6%)	11 (7.1%)	0.251
21. I felt watchful and on-guard.			
. - none	20 (14.5%)	11 (7.1%)	
-not much	24 (17.4%)	13 (8.4%)	
-moderately	25 (18.1%)	51 (32.9%)	
-enough	50 (36.2%)	51 (32.9%)	
-extremely	19 (13.8%)	29 (18.7%)	0.003*
22. I tried not to talk about it. them.			
- none	56 (40.6%)	41 (26.5%)	
-not much	35 (25.4%)	35 (22.6%)	
-moderately	23 (16.7%)	45 (29%)	
-enough	15 (10.4%)	25 (16.1%)	
-extremely	9 (6.5%)	9 (5.8%)	0.027*

supplementary

BDI TEST

supplementary

Table S2. relationship between the menstrual cycle and the impact of the pandemic as a traumatic event.
A, B: relationship with the altered duration of the menstrual cycle; C: Amenorrhea; D: Change Weight; E: Premenstrual syndrome

A **IN THE LAST YEAR ALTERATION OF MENSTRUAL CYCLE**

PATIENTS n.293 (100%)	No (183 / 62.5%)	Increse of interval (73/24.9%)	Reduction of interval (37/12.6%)	P
QUESTION 1				
do not feel sad.	89(48.6%)	16(21.9%)	10 (27%)	
feel sad	78(42.6%)	39(53.4%)	22 (59.5%)	
-I am sad all the time and I can't snap out of it	15(8.2%)	15(20.5%)	3 (8.1%)	
I am so sad and unhappy that I can't stand it	1(0.5%)	3(4.1%)	2 (5.4%)	<0.001*
QUESTION 2 -				
I am not particularly discouraged about the future				
I feel discouraged about the future.	88(48.1%)	29(39.7%)	14(37.8%)	
I feel I have nothing to look forward to.	75(41.0%)	36(49.3%)	14(37.8%)	
I feel the future is hopeless and that things	15(8.2%)	6(8.2%)	6(16.2%)	
cannot improve	5(2.7%)	2(2.7%)	3(8.1%)	0.291
QUESTION 3				
I do not feel like a failure				
I feel I have failed more than the average person.	126(68.9%)	47(64.4%)	20(54.1%)	
As I look back on my life, all I can see is a lot of	38(20.8%)	22(30.1%)	8(21.6%)	
failures.	14(7.7%)	2(2.7%)	2(5.4%)	
I feel I am a complete failure as a person.	5(2.7%)	2(2.7%)	7(18.9%)	<0.001*
QUESTION 4				
I get as much satisfaction out of things as I used to.				
I don't enjoy things the way I used to.	63(34.4%)	19(26%)	10(27%)	
I don't get real satisfaction out of anything	90(49.2%)	41(56.2%)	20(54.1%)	
anymore.	11(6%)	9(12.3%)	3(8.1%)	
I am dissatisfied or bored with everything	19(10.4%)	4(5.5%)	4(10.8%)	0.441
QUESTION 5				
I don't feel particularly guilty	106(57.9%)	36(49.3%)	14(37.8%)	
I feel guilty a good part of the time.	49(26.8%)	23(31.5%)	13(35.1%)	
I feel quite guilty most of the time.	21(11.5%)	10(13.7%)	8(21.6%)	
I feel guilty all of the time.	7(3.8%)	4(5.5%)	2(5.4%)	0.383
QUESTION 6				
I don't feel I am being punished.	134(73.2%)	52(71.2%)	22(59.5%)	
I feel I may be punished.	25(13.7%)	6(8.2%)	10(27%)	
I expect to be punished.	6(3.3%)	7(9.6%)	2(5.4%)	
feel I am being punished.	18(9.8%)	8(11%)	3(8.1%)	0.079
QUESTION 7				
I don't feel disappointed in myself.	123(67.2%)	40(54.8%)	18(48.6%)	
I am disappointed in myself.	48(26.2%)	27(37%)	17(45.9%)	
I am disgusted with myself.	6(3.3%)	5(6.8%)	1(2.7%)	
I hate myself.	6(3.3%)	1(1.4%)	1(2.7%)	0.134
QUESTION 8				
I don't feel I am any worse than anybody else.				
I am critical of myself for my weaknesses or mistakes.	32(17.5%)	7(9.6%)	3(8.1%)	
I blame myself all the time for my faults.	134(73.2%)	57(78.1%)	30(81.1%)	
I blame myself for everything bad that	14(7.7%)	5(6.8%)	2(5.4%)	
happens.	3(1.6%)	4(5.5%)	2(5.4%)	0.299
QUESTION 9				
I don't have any thoughts of killing myself.				
I have thoughts of killing myself, but I would	171(93.4%)	64(87.7%)	33(89.2%)	
not carry them out.	11(6%)	8(11%)	3(8.1%)	
I would like to kill myself.	0	1(1.4%)	1(2.7%)	
I would kill myself if I had the chance.	1(0.5%)	0	0	0.369

QUESTION 10				
I don't cry any more than usual.				
I cry more now than I used to.	104(56.8%)	28(38.4%)	22(59.5%)	
I cry all the time now.	67(36.6%)	38(52.1%)	11(29.7%)	
used to be able to cry, but now I can't cry even	5(2.7%)	1(1.4%)	1(2.7%)	
though I want to	7(3.8%)	6(8.2%)	3(8.1%)	0.096
QUESTION 11				
I am no more irritated by things than I ever				
was.	59(32.2%)			
I am slightly more irritated now than usual.	83(45.4%)	19(26%)	16(43.2%)	
I am quite annoyed or irritated a good deal of	39(21.3%)	29(39.7%)	14(37.8%)	
the time.	2(1.1%)	25(34.2%)	6(16.2%)	
I feel irritated all the time.		0	1(2.7%)	0.153
QUESTION 12				
I have not lost interest in other people.				
I am less interested in other people than I used	89(48.6%)	25(34.2%)	20(54.1%)	
to be.	77(42.1%)	32(43.8%)	12(32.4%)	
I have lost most of my interest in other people.	16(8.7%)	15(20.5%)	5(13.5%)	
I have lost all of my interest in other people	1(0.5%)	1(1.4%)	0	0.101
QUESTION 13				
I make decisions about as well as I ever could.				
I put off making decisions more than I used to.	117(63.9%)	38(52.1%)	20(54.1%)	
I have greater difficulty in making decisions	37(20.2%)	21(28.8%)	12(32.4%)	
more than I used to.	25(13.7%)	12(16.4%)	3(8.1%)	
I can't make decisions at all anymore.	4(2.2%)	2(2.7%)	2(5.4%)	0.343
QUESTION 14				
I don't feel that I look any worse than I used to.				
I am worried that I am looking old or				
unattractive.	104(56.8%)	29(39.7%)	16(43.2%)	
I feel there are permanent changes in my	48(26.2%)	16(21.9%)	5(13.5%)	
appearance that make me look unattractive	15(8.2%)	16(21.9%)	13(35.1%)	
believe that I look ugly.	16(8.7%)	12(16.4%)	3(8.1%)	<0.001*
QUESTION 15				
I can work about as well as before.				
It takes an extra effort to get started at doing	69(37.7%)	16(21.9%)	12(32.4%)	
something.	80(43.7%)	32(43.8%)	16(43.2%)	
I have to push myself very hard to do anything.	33(18%)	25(34.2%)	7(18.9%)	
I can't do any work at all.	1(0.5%)	0	2(5.4%)	0.005*
QUESTION 16				
I can sleep as well as usual.				
I don't sleep as well as I used to.				
I wake up 1-2 hours earlier than usual and find	89(48.6%)	16(21.9%)	10(27%)	
it hard to get back to sleep.	69(37.7%)	31(42.5%)	18(48.6%)	
I wake up several hours earlier than I used to	20(10.9%)	18(24.7%)	7(18.9%)	
and cannot get back to sleep.	5(2.7%)	8(11%)	2(5.4%)	<0.001*
QUESTION 17				
I don't get more tired than usual.	49(26.8%)	14(19.2%)	7(18.9%)	
I get tired more easily than I used to.	110(60.1%)	46(63%)	22(59.5%)	
I get tired from doing almost anything.	21(11.5%)	9(12.3%)	4(10.8%)	
I am too tired to do anything.	3(1.6%)	4(5.5%)	4(10.8%)	0.147
			37(50.7%)	
QUESTION 18			29(39.7%)	
-My appetite is no worse than usual.	141(77%)	37(50.7%)		
My appetite is not as good as it used to be.	35(19.1%)	29(39.7%)	6(8.2%)	
My appetite is much worse now.	7(3.8%)	6(8.2%)	1(1.4%)	
have no appetite at all anymore	0	1(1.4%)		<0.001*
QUESTION 19	145(79.2%)			
I haven't lost much weight, if any, lately.	31(16.9%)	49(67.1%)	29(78.4%)	
I have lost more than five pounds.	6(3.3%)	17(23.3%)	4(10.8%)	
I have lost more than ten pounds.	1(0.5%)	4(5.5%)	2(5.4%)	
I have lost more than fifteen pounds.		3(4.1%)	2(5.4%)	0.137
QUESTION 20				
I am no more worried about my health than				
usual.				
I am worried about physical problems like				
aches, pains, upset stomach, or constipation.				
I am very worried about physical problems and	124(67.8%)	42(57.5%)	25(67.6%)	
it's hard to think of much else.	51(27.9%)	23(31.5%)	11(29.7%)	
I am so worried about my physical problems	6(3.3%)	7(9.6%)	1(2.7%)	
that I cannot think of anything else.	2(1.1%)	1(1.4%)	0	0.388

QUESTION 21<br>. I have not<br>noticed any recent change in my interest in sex.<br>I am less interested in sex than I used to be.<br>I have almost no interest in sex.<br>I have lost interest in sex completely.	126(68.9%)<br>49(26.8%)<br>5(2.7%)<br>3(1.6%)	42(57.5%)<br>26(35.6%)<br>4(5.5%)<br>1(1.4%)	24(64.9%)<br>10(27%)<br>2(5.4%)<br>1(2.7%)	0.662
22. I tried not to talk about it. them.<br>- none<br>-not much<br>-moderately<br>-enough<br>-extremely	71 (38.8%)<br>44 (24.0%)<br>37 (20.2%)<br>20 (10.9%)<br>11 (6.0%)	19 (26%)<br>11 (15.1%)<br>24 (32.9%)<br>14 (19.2%)<br>5 (6.8%)	7 (18.9%)<br>15 (40.5%)<br>7 (18.9%)<br>6 (16.2%)<br>2 (5.4%)	0.017*

B IN THE LAST YEAR HOW OFTEN ALTERATION OF MENSTRUAL CYCLE

PATIENTS n.293 (100%)	21-35 D (260 / 88.7%)	>35 D (29/9.9%)	<21D (4/1.4%)	P
QUESTION 1<br>do not feel sad.<br>feel sad<br>-I am sad all the time and I can't snap out of it<br>I am so sad and unhappy that I can't stand it	107 (41.2%)<br>121(46.5%)<br>29(11.2%)<br>3(1.2%)	8(27.6%)<br>14(48.3%)<br>4(13.8%)<br>3(10.3%)	0<br>4 (100%)<br>0<br>0	0.011*
QUESTION 3<br>I do not feel like a failure<br>I feel I have failed more than the average person.<br>As I look back on my life, all I can see is a lot of<br>failures.<br>I feel I am a complete failure as a person.	173(66.5%)<br>62(23.8%)<br>15(5.8%)<br>10(3.8%)	19(65.5%)<br>6(20.7%)<br>2(6.9%)<br>2(6.9%)	1(25%)<br>0<br>1(25%)<br>2(50%)	0.001*
QUESTION 5<br>I don't feel particularly guilty<br>I feel guilty a good part of the time.<br>I feel quite guilty most of the time.<br>I feel guilty all of the time.	143(55%)<br>73(28.1%)<br>33(12.7%)<br>11(4.2%)	13(44.8%)<br>11(37.9%)<br>3(10.3%)<br>2(6.9%)	0<br>1(25%)<br>3(75%)<br>0	0.015*
QUESTION 9<br>I don't have any thoughts of killing myself.<br>I have thoughts of killing myself, but I would not<br>carry them out.<br>I would like to kill myself.<br>I would kill myself if I had the chance.	239(91.9%)<br>20(7.7%)<br>0<br>1(0.4%)	25(86.2%)<br>2(6.9%)<br>2(6.9%)<br>0	4(100%)<br>0<br>0<br>0	0.005*
QUESTION 12<br>I have not lost interest in other people.<br>I am less interested in other people than I used to be.<br>I have lost most of my interest in other people.<br>I have lost all of my interest in other people	126(48.5%)<br>106(40.8%)<br>26(10%)<br>2(0.8%)	5(17.2%)<br>14(48.3%)<br>10(34.5%)<br>0	3(75%)<br>1(25%)<br>0<br>0	0.002*
QUESTION 14<br>I don't feel that I look any worse than I used to.<br>I am worried that I am looking old or unattractive.<br>I feel there are permanent changes in my appearance<br>that make me look unattractive<br>believe that I look ugly.	69(37.7%)<br>80(43.7%)<br>33(18%)<br>1(0.5%)	16(21.9%)<br>32(43.8%)<br>25(34.2%)<br>0	12(32.4%)<br>16(43.2%)<br>7(18.9%)<br>2(5.4%)	0.005*
QUESTION 16<br>I can sleep as well as usual.<br>I don't sleep as well as I used to.<br>I wake up 1-2 hours earlier than usual and find it hard<br>to get back to sleep.<br>I wake up several hours earlier than I used to and<br>cannot get back to sleep.	108(41.5%)<br>105(40.4%)<br>34(13.1%)<br>13(5%)	5(17.2%)<br>12(41.4%)<br>10(34.5%)<br>2(6.9%)	2(50 %)<br>1(25%)<br>1(25%)<br>0	0.048*
QUESTION 20<br>I am no more worried about my health than usual.<br>I am worried about physical problems like aches,<br>pains, upset stomach, or constipation.<br>I am very worried about physical problems and it's<br>hard to think of much else.<br>I am so worried about my physical problems that I<br>cannot think of anything else.	170(65.4%)<br>78(30%)<br>11(4.2%)<br>1(0.4%)	20(69%)<br>4(13.8%)<br>3(10.3%)<br>2(6.9%)	1(25%)<br>3(75)<br>0<br>0	0.003*
QUESTION 21<br>. I have not noticed any<br>recent change in my interest in sex.<br>I am less interested in sex than I used to be.<br>I have almost no interest in sex.<br>I have lost interest in sex completely.	177(68.1%)<br>72(27.7%)<br>9(3.5%)<br>2(0.8%)	12(41.4%)<br>12(41.4%)<br>2(6.9%)<br>3(10.3%)	3(75%)<br>1(25%)<br>0<br>0	0.003*

C

	IN THE LAST YEAR AMENORRHEA		
**PATIENTS n.293 (100%)**	**NO 270 (92.2%)**	**YES 23 (7.8%)**	**P**
QUESTION 1			
I do not feel sad.	110(40.7%)	5(21.7%)	
I feel sad	127(47%)	12(52.2%)	
I am sad all the time and I can't snap out of it	30(11.1%)	3(13%)	
I am so sad and unhappy that I can't stand it	3(1.1%)	3(13%)	<0.001*
QUESTION 2			
I am not particularly discouraged about the future			
I feel discouraged about the future.	123(45.6%)	8(34.8%)	
I feel I have nothing to look forward to.	115(42.6%)	10(43.5%)	
I feel the future is hopeless and that things cannot	23(8.5%)	4(17.4%)	
improve	9(3.3%)	1(4.3%)	0.488
QUESTION 3			
I do not feel like a failure	180(66.7%)	13(56.5%)	
I feel I have failed more than the average person.	61(22.6%)	7(30.4%)	
As I look back on my life, all I can see is a lot of failures.	17(6.3%)	1(4.3%)	
I feel I am a complete failure as a person.	12(4.4%)	2(8.7%)	0.609
QUESTION 4			
I get as much satisfaction out of things as I used to.	87(32.2%)	5(21.7%)	
I don't enjoy things the way I used to.	139(51.5%)	12(52.2%)	
I don't get real satisfaction out of anything anymore.	19(7%)	4(17.4%)	
I am dissatisfied or bored with everything	25(9.3%)	2(8.7%)	0.302
QUESTION 5			
I don't feel particularly guilty	142(52.6%)	14(60.9%)	
I feel guilty a good part of the time.	80(29.6%)	5(21.7%)	
I feel quite guilty most of the time.	36(13.3%)	3(13%)	
I feel guilty all of the time.	12(4.4%)	1(4.3%)	0.863
QUESTION 6			
I don't feel I am being punished.	195(72.2%)	13(56.5%)	
I feel I may be punished.	35(13%)	6(26.1%)	
I expect to be punished.	14(5.2%)	1(4.3%)	
feel I am being punished.	26(9.6%)	3(13%)	0.305
QUESTION 7			
I don't feel disappointed in myself.	173(64.1%)	8(34.8%)	
I am disappointed in myself.	79(29.3%)	13(56.5%)	
I am disgusted with myself.	10(3.7%)	2(8.7%)	
I hate myself.	8(3%)	0	0.019*
QUESTION 8			
I don't feel I am any worse than anybody else.	39(14.4%)	3(13%)	
I am critical of myself for my weaknesses or mistakes.	204(75.6%)	17(73.9%)	
I blame myself all the time for my faults.	18(6.7%)	3(13%)	
I blame myself for everything bad that happens.	9(3.3%)	0	0.571
QUESTION 9	250(92.6%)		
I don't have any thoughts of killing myself.	18(6.7%)	18(78.3%)	
I have thoughts of killing myself, but I would not carry them out.	1(0.4%)	4(17.4%)	
I would like to kill myself.	1(0.4%)	1(4.3%)	
I would kill myself if I had the chance.		0	0.033*
QUESTION 10			
I don't cry any more than usual.	144(53.3%)	10(43.5%)	
I cry more now than I used to.	108(40.0%)	8(34.8%)	
I cry all the time now.	6(2.2%)	1(4.3%)	
used to be able to cry, but now I can't cry even though I want to	12(4.4%)	4(17.4%)	0.591
QUESTION 11			
I am no more irritated by things than I ever was.	87(32.2%)	7(30.4%)	
I am slightly more irritated now than usual.	118(43.7%)	8(34.8%)	
I am quite annoyed or irritated a good deal of the time.	63(23.3%)	7(30.4%)	
I feel irritated all the time.	2(0.7%)	1(4.3%)	0.314
QUESTION 12			
I have not lost interest in other people.	125(46.3%)	9(39.1%)	
I am less interested in other people than I used to be.	114(42.2%)	7(30.4%)	
I have lost most of my interest in other people.	29(10.7%)	7(30.4%)	
I have lost all of my interest in other people	2(0.7%)	0	0.050*
QUESTION 13			
I make decisions about as well as I ever could.	166(61.5%)	9(39.1%)	
I put off making decisions more than I used to.	62(23%)	8(34.8%)	
I have greater difficulty in making decisions more than I used to.	36(13.3%)	4(17.4%)	
I can't make decisions at all anymore.	6(2.2%)	2(8.7%)	0.089

QUESTION 14<br>I don't feel that I look any worse than I used to.<br>I am worried that I am looking old or unattractive.<br>I feel there are permanent changes in my appearance that make me look unattractive<br>believe that I look ugly.	142(52.6%)<br>64(23.7%)<br>37(13.7%)<br>27(10%)	7(30.4%)<br>5(21.7%)<br>7(30.4%)<br>4(17.4%)	0.068
QUESTION 15<br>I can work about as well as before.<br>It takes an extra effort to get started at doing something.<br>I have to push myself very hard to do anything.<br>I can't do any work at all.	92(34.1%)<br>119(44.1%)<br>57(21.1%)<br>2(0.7%)	5(21.7%)<br>9(39.1%)<br>8(34.8%)<br>1(4.3%)	0.134
QUESTION 16<br>I can sleep as well as usual.<br>I don't sleep as well as I used to.<br>I wake up 1-2 hours earlier than usual and find it hard to get back to sleep.<br>I wake up several hours earlier than I used to and cannot get back to sleep.	112(41.5%)<br>114(42.2%)<br>32(11.9%)<br>12(4.4%)	3(13.0%)<br>4(17.4%)<br>13(56.5%)<br>3(13%)	<0.001*
QUESTION 17<br>I don't get more tired than usual.<br>I get tired more easily than I used to.<br>I get tired from doing almost anything.<br>I am too tired to do anything.	65(24.1%)<br>164(60.7%)<br>30(11.1%)<br>11(4.1%)	5(21.7%)<br>14(60.9%)<br>4(17.4%)<br>0	0.636
QUESTION 18<br>-My appetite is no worse than usual.<br>My appetite is not as good as it used to be.<br>My appetite is much worse now.<br>have no appetite at all anymore	188(69.6%)<br>67(24.8%)<br>14(5.2%)<br>1(0.4%)	11(47.8%)<br>12(52.2%)<br>0<br>0	0.034*
QUESTION 19<br>I haven't lost much weight, if any, lately.<br>I have lost more than five pounds.<br>I have lost more than ten pounds.<br>I have lost more than fifteen pounds.	209(77.4%)<br>45(16.7%)<br>11(4.1%)<br>5(1.9%)	14(60.9%)<br>7(30.4%)<br>1(4.3%)<br>1(4.3%)	0.299
QUESTION 20<br>I am no more worried about my health than usual.<br>I am worried about physical problems like aches, pains, upset stomach, or constipation.<br>I am very worried about physical problems and it's hard to think of much else.<br>I am so worried about my physical problems that I cannot think of anything else.	178(65.9%)<br>78(28.9%)<br>12(4.4%)<br>2(0.7%)	13(56.5%)<br>7(30.4%)<br>2(8.7%)<br>1(4.3%)	0.284
QUESTION 21<br>I have not noticed any recent change in my interest in sex.<br>I am less interested in sex than I used to be.<br>I have almost no interest in sex.<br>I have lost interest in sex completely.	178(65.9%)<br>80(29.6%)<br>8(3%)<br>4(1.5%)	14(60.9%)<br>5(21.7%)<br>3(13%)<br>1(4.3%)	0.063

D		IN THE LAST YEAR CHANGE WEIGHT		
**PATIENTS n.293 (100%)**	**No (132 / 45.1%)**	**Increase (86/29.4%)**	**Reduction (75/25.6%)**	**P**
QUESTION 1<br>Do not feel sad.<br>I feel sad.<br>I am sad all the time and I can't snap out of it.<br>I am so sad and unhappy that I can't stand it.	51(38.6%)<br>66(50%)<br>15(11.4%)<br>0	39(45.3%)<br>34(39.5%)<br>11(12.8%)<br>2(2.3%)	25(33.3%)<br>39(52%)<br>7(9.3%)<br>4(5.3%)	0.114
QUESTION 2<br>I am not particularly discouraged about the future<br>I feel discouraged about the future.<br>I feel I have nothing to look forward to.<br>I feel the future is hopeless and that things cannot improve	56(42.4%)<br>61(46.2%)<br>9(6.8%)<br>6(4.5%)	42(48.8%)<br>30(34.9%)<br>11(12.8%)<br>3(3.5%)	33(44%)<br>34(45.3%)<br>7(9.3%)<br>1(1.3%)	0.459
QUESTION 3<br>I do not feel like a failure<br>I feel I have failed more than the average person.<br>As I look back on my life, all I can see is a lot of failures.	88(66.7%)<br>32(24.2%)<br>5(3.8%)<br>7(5.3%)	53(61.6%)<br>20(23.3%)<br>8(9.3%)<br>5(5.8%)	52(69.3%)<br>16(21.3%)<br>5(6.7%)<br>2(2.7%)	0.656

I feel I am a complete failure as a person.				
QUESTION 4<br>I get as much satisfaction out of things as I used to.<br>I don't enjoy things the way I used to.<br>I don't get real satisfaction out of anything anymore.<br>I am dissatisfied or bored with everything	44(33.3%)<br>67(50.8%)<br>8(6.1%)<br>13(9.8%)	25(29.1%)<br>44(51.2%)<br>11(12.8%)<br>6(7%)	23(30.7%)<br>40(53.3%)<br>4(5.3%)<br>8(10.7%)	0.557
QUESTION 5<br>I don't feel particularly guilty<br>I feel guilty a good part of the time.<br>I feel quite guilty most of the time.<br>I feel guilty all of the time.	72(54.5%)<br>36(27.3%)<br>18(13.6%)<br>6(4.5%)	45(52.3%)<br>23(26.7%)<br>13(15.1%)<br>5(5.8%)	39(52%)<br>26(34.7%)<br>8(10.7%)<br>2(2.7%)	0.844
QUESTION 6<br>I don't feel I am being punished.<br>I feel I may be punished.<br>I expect to be punished.<br>feel I am being punished.	96(72.7%)<br>16(12.1%)<br>7(5.3%)<br>13(9.8%)	57(66.3%)<br>12(14%)<br>7(8.1%)<br>10(11.6%)	55(73.3%)<br>13(17.3%)<br>1(1.3%)<br>6(8%)	0.483
QUESTION 7<br>I don't feel disappointed in myself.<br>I am disappointed in myself.<br>I am disgusted with myself.<br>I hate myself.	85(64.4%)<br>38(28.8%)<br>5(3.8%)<br>4(3%)	44(51.2%)<br>37(43%)<br>3(3.5%)<br>2(2.3%)	52(69.3%)<br>17(22.7%)<br>4(5.3%)<br>2(2.7%)	0.191
QUESTION 8<br>I don't feel I am any worse than anybody else.<br>I am critical of myself for my weaknesses or mistakes.<br>I blame myself all the time for my faults.<br>I blame myself for everything bad that happens.	17(12.9%)<br>98(74.2%)<br>11(8.3%)<br>6(4.5%)	12(14%)<br>68(79.1%)<br>5(5.8%)<br>1(1.2%)	13(17.3%)<br>55(73.3%)<br>5(6.7%)<br>2(2.7%)	0.759
QUESTION 9<br>I don't have any thoughts of killing myself.<br>I have thoughts of killing myself, but I would not carry them out.<br>I would like to kill myself.<br>I would kill myself if I had the chance.	121(91.7%)<br>11(8.3%)<br>0<br>0	76(88.4%)<br>7(8.1%)<br>2(2.3%)<br>1(1.2%)	71(94.7%)<br>4(5.3%)<br>0<br>0	0.236
QUESTION 10<br>I don't cry any more than usual.<br>I cry more now than I used to.<br>I cry all the time now.<br>used to be able to cry, but now I can't cry even though I want to	73(55.3%)<br>52(39.4%)<br>2(1.5%)<br>5(3.8%)	46(53.5%)<br>31(36%)<br>2(2.3%)<br>7(8.1%)	35(46.7%)<br>33(44%)<br>3(4%)<br>4(5.3%)	0.624
QUESTION 11<br>I am no more irritated by things than I ever was.<br>I am slightly more irritated now than usual.<br>I am quite annoyed or irritated a good deal of the time.<br>I feel irritated all the time.	47(35.6%)<br>57(43.2%)<br>27(20.5%)<br>1(0.8%)	28(32.6%)<br>37(43%)<br>19(22.1%)<br>2(2.3%)	19(25.3%)<br>32(42.7%)<br>24(32%)<br>0	0.349
QUESTION 12<br>I have not lost interest in other people.<br>I am less interested in other people than I used to be.<br>I have lost most of my interest in other people.<br>I have lost all of my interest in other people	69(52.3%)<br>48(36.4%)<br>15(11.4%)<br>0	37(43%)<br>40(46.5%)<br>7(8.1%)<br>2(2.3%)	28(37.3%)<br>33(44%)<br>14(18.7%)<br>0	0.050*
QUESTION 13<br>I make decisions about as well as I ever could.<br>I put off making decisions more than I used to.<br>I have greater difficulty in making decisions more than I used to.<br>I can't make decisions at all anymore.	84(63.6%)<br>31(23.5%)<br>14(10.6%)<br>3(2.3%)	44(51.2%)<br>24(27.9%)<br>16(18.6%)<br>2(2.3%)	47(62.7%)<br>15(20%)<br>10(13.3%)<br>3(4%)	0.468
QUESTION 14<br>I don't feel that I look any worse than I used to.<br>I am worried that I am looking old or unattractive.<br>I feel there are permanent changes in my appearance that make me look unattractive.<br>believe that I look ugly.	78(59.1%)<br>25(18.9%)<br>11(8.3%)<br>18(13.6%)	30(34.9%)<br>27(31.4%)<br>23(26.7%)<br>6(7%)	41(54.7%)<br>17(22.7%)<br>10(13.3%)<br>7(9.3%)	< 0.001*

QUESTION 15<br>I can work about as well as before.<br>It takes an extra effort to get started at doing something.<br>I have to push myself very hard to do anything.<br>I can't do any work at all.	53(40.2%)<br>56(42.4%)<br>23(17.4%)<br>0	21(24.4%)<br>38(44.2%)<br>25(29.1%)<br>2(2.3%)	23(30.7%)<br>34(45.3%)<br>17(22.7%)<br>1(1.3%)	0.116
QUESTION 16<br>I can sleep as well as usual.<br>I don't sleep as well as I used to.<br>I wake up 1-2 hours earlier than usual and find it hard to get back to sleep.<br>I wake up several hours earlier than I used to and cannot get back to sleep.	63(47.7%)<br>51(38.6%)<br>12(9.1%)<br>6(4.5%)	29(33.7%)<br>35(40.7%)<br>18(20.9%)<br>4(4.7%)	23(30.7%)<br>32(42.7%)<br>15(20%)<br>5(6.7%)	0.079
QUESTION 17<br>I don't get more tired than usual.<br>I get tired more easily than I used to.<br>I get tired from doing almost anything.<br>I am too tired to do anything.	34(25.8%)<br>82(62.1%)<br>12(9.1%)<br>4(3%)	17(19.8%)<br>52(60.5%)<br>11(12.8%)<br>6(7%)	19(25.3%)<br>44(58.7%)<br>11(14.7%)<br>1(1.3%)	0.409
QUESTION 18<br>My appetite is no worse than usual.<br>My appetite is not as good as it used to be.<br>My appetite is much worse now.<br>have no appetite at all anymore	104(78.8%)<br>23(17.4%)<br>5(3.8%)<br>0	56(65.1%)<br>23(26.7%)<br>7(8.1%)<br>0	39(52%)<br>33(44%)<br>2(2.7%)<br>1(1.3%)	< 0.001*
QUESTION 19<br>I haven't lost much weight, if any, lately.<br>I have lost more than five pounds.<br>I have lost more than ten pounds.<br>I have lost more than fifteen pounds.	118(89.4%)<br>13(9.8%)<br>0<br>1(0.8%)	78(90.7%)<br>5(5.8%)<br>2(2.3%)<br>1(1.2%)	27(36%)<br>34(45.3%)<br>10(13.3%)<br>4(5.3%)	< 0.001*
QUESTION 20<br>I am no more worried about my health than usual.<br>I am worried about physical problems like aches, pains, upset stomach, or constipation.<br>I am very worried about physical problems and it's hard to think of much else.<br>I am so worried about my physical problems that I cannot think of anything else.	86(65.2%)<br>36(27.3%)<br>8(6.1%)<br>2(1.5%)	59(68.6%)<br>26(30.2%)<br>1(1.2%)<br>0	46(61.3%)<br>23(30.7%)<br>5(6.7%)<br>1(1.3%)	0.518
QUESTION 21<br>I have not noticed any recent change in my interest in sex.<br>I am less interested in sex than I used to be.<br>I have almost no interest in sex.<br>I have lost interest in sex completely.	85(64.4%)<br>40(30.3%)<br>4(3%)<br>3(2.3%)	57(66.3%)<br>25(29.1%)<br>3(3.5%)<br>1(1.2%)	50(66.7%)<br>20(26.7%)<br>4(5.3%)<br>1(1.3%)	0.964

E	IN THE LAST YEAR PREMENSTRUAL SYNDROME		
**PATIENTS n.293 (100%)**	**NO (138 / 47.1%)**	**YES (155/52.9%)**	**P**
QUESTION 1<br>do not feel sad.<br>feel sad<br>-I am sad all the time and I can't snap out of it<br>I am so sad and unhappy that I can't stand it	64(46.4%)<br>65(47.1%)<br>7(5.1%)<br>2(1.4%)	51(32.9%)<br>74(47.7%)<br>26(16.8%)<br>4(2.6%)	0.005*
QUESTION 2<br>I am not particularly discouraged about the future<br>I feel discouraged about the future.<br>I feel I have nothing to look forward to.<br>I feel the future is hopeless and that things cannot improve	65(47.1%)<br>61(44.2%)<br>11(8%)<br>1(0.7%)	66(42.6%)<br>64(41.3%)<br>16(10.3%)<br>9(5.8%)	0.092
QUESTION 3<br>I do not feel like a failure<br>I feel I have failed more than the average person.<br>As I look back on my life, all I can see is a lot of failures.<br>I feel I am a complete failure as a person.	98(71%)<br>30(21.7%)<br>5(3.6%)<br>5(3.6%)	95(61.3%)<br>38(24.5%)<br>13(8.4%)<br>9(5.8%)	0.194

QUESTION 4<br>I get as much satisfaction out of things as I used to.<br>I don't enjoy things the way I used to.<br>I don't get real satisfaction out of anything anymore.<br>I am dissatisfied or bored with everything	46(33.3%)<br>75(54.3%)<br>4(2.9%)<br>13(9.4%)	46(29.7%)<br>76(49%)<br>19(12.3%)<br>14(9%)	0.031*
QUESTION 5<br>I don't feel particularly guilty<br>I feel guilty a good part of the time.<br>I feel quite guilty most of the time.<br>I feel guilty all of the time.	81(58.7%)<br>39(28.3%)<br>11(8%)<br>7(5.1%)	75(48.4%)<br>46(29.7%)<br>28(18.1%)<br>6(3.9%)	0.062
QUESTION 6<br>I don't feel I am being punished.<br>I feel I may be punished.<br>I expect to be punished.<br>feel I am being punished.	98(71%)<br>24(17.4%)<br>3(2.2%)<br>13(9.4%)	110(71%)<br>17(11%)<br>12(7.7%)<br>16(10.3%)	0.085
QUESTION 7<br>I don't feel disappointed in myself.<br>I am disappointed in myself.<br>I am disgusted with myself.<br>I hate myself.	95(68.8%)<br>37(26.8%)<br>3(2.2%)<br>3(2.2%)	86(55.5%)<br>55(35.5%)<br>9(5.8%)<br>5(3.2%)	0.089
QUESTION 8<br>I don't feel I am any worse than anybody else.<br>I am critical of myself for my weaknesses or mistakes.<br>I blame myself all the time for my faults.<br>I blame myself for everything bad that happens.	16(11.6%)<br>109(79%)<br>10(7.2%)<br>3(2.2%)	26(16.8%)<br>112(72.3%)<br>11(7.1%)<br>6(3.9%)	0.477
QUESTION 9<br>I don't have any thoughts of killing myself.<br>I have thoughts of killing myself, but I would not carry them out.<br>I would like to kill myself.<br>I would kill myself if I had the chance.	128(92.8%)<br>8(5.8%)<br>1(0.7%)<br>1(0.7%)	140(90.3%)<br>14(9%)<br>1(0.6%)<br>0	0.533
QUESTION 10<br>I don't cry any more than usual.<br>I cry more now than I used to.<br>I cry all the time now.<br>used to be able to cry, but now I can't cry even though I want to	82(59.4%)<br>46(33.3%)<br>2(1.4%)<br>8(5.8%)	72(46.5%)<br>70(45.2%)<br>5(3.2%)<br>8(5.2%)	0.115
QUESTION 11<br>I am no more irritated by things than I ever was.<br>I am slightly more irritated now than usual.<br>I am quite annoyed or irritated a good deal of the time.<br>I feel irritated all the time.	57(41.3%)<br>52(37.7%)<br>27(19.6%)<br>2(1.4%)	37(23.9%)<br>74(47.7%)<br>43(27.7%)<br>1(0.6%)	0.011*
QUESTION 12<br>I have not lost interest in other people.<br>I am less interested in other people than I used to be.<br>I have lost most of my interest in other people.<br>I have lost all of my interest in other people	64(46.4%)<br>54(39.1%)<br>20(14.5%)<br>0	70(45.2%)<br>67(43.2%)<br>16(10.3%)<br>2(1.3%)	0.371
QUESTION 13<br>I make decisions about as well as I ever could.<br>I put off making decisions more than I used to.<br>I have greater difficulty in making decisions more than I used to.<br>I can't make decisions at all anymore.	95(68.8%)<br>25(18.1%)<br>15(10.9%)<br>3(2.2%)	80(51.6%)<br>45(29%)<br>25(16.1%)<br>5(3%)	0.029*
QUESTION 14<br>I don't feel that I look any worse than I used to.<br>I am worried that I am looking old or unattractive.<br>I feel there are permanent changes in my appearance that make me look unattractive<br>believe that I look ugly.	80(58%)<br>33(23.9%)<br>12(8.7%)<br>13(9.4%)	69(44.5%)<br>36(23.2%)<br>32(20.6%)<br>18(11.6%)	0.020*
QUESTION 15<br>I can work about as well as before.<br>It takes an extra effort to get started at doing something.<br>I have to push myself very hard to do anything.<br>I can't do any work at all.	55(39.9%)<br>54(39.1%)<br>28(20.3%)<br>1(0.7%)	42(27.1%)<br>74(47.7%)<br>37(23.9%)<br>2(1.3%)	0.140

QUESTION 16<br>I can sleep as well as usual.<br>I don't sleep as well as I used to.<br>I wake up 1-2 hours earlier than usual and find it hard to get back to sleep.<br>I wake up several hours earlier than I used to and cannot get back to sleep.	62(44.9%)<br>53(38.4%)<br>20(14.5%)<br>3(2.2%)	53(34.2%)<br>65(41.9%)<br>25(16.1%)<br>12(7.7%)	0.075
QUESTION 17<br>I don't get more tired than usual.<br>I get tired more easily than I used to.<br>I get tired from doing almost anything.<br>I am too tired to do anything.	42(30.4%)<br>81(58.7%)<br>11(8%)<br>4(2.9%)	28(18.1%)<br>97(62.6%)<br>23(14.8%)<br>7(4.5%)	0.040*
QUESTION 18<br>My appetite is no worse than usual.<br>My appetite is not as good as it used to be.<br>My appetite is much worse now.<br>have no appetite at all anymore	100(72.5%)<br>33(23.9%)<br>5(3.6%)<br>0	99(63.9%)<br>46(29.7%)<br>9(5.8%)<br>1(0.6%)	0.346
QUESTION 19<br>I haven't lost much weight, if any, lately.<br>I have lost more than five pounds.<br>I have lost more than ten pounds.<br>I have lost more than fifteen pounds.	99(71.7%)<br>28(20.3%)<br>6(4.3%)<br>5(3.6%)	124(80%)<br>24(15.5%)<br>6(3.9%)<br>1(0.6%)	0.186
QUESTION 20<br>I am no more worried about my health than usual.<br>I am worried about physical problems like aches, pains, upset stomach, or constipation.<br>I am very worried about physical problems and it's hard to think of much else.<br>I am so worried about my physical problems that I cannot think of anything else.	98(71%)<br>35(25.4%)<br>4(2.9%)<br>1(0.7%)	93(60%)<br>50(32.3%)<br>10(6.5%)<br>2(1.3%)	0.194
QUESTION 21<br>I have not noticed any recent change in my interest in sex.<br>I am less interested in sex than I used to be.<br>I have almost no interest in sex.<br>I have lost interest in sex completely.	96(69.6%)<br>36(26.1%)<br>4(2.9%)<br>2(1.4%)	96(61.9%)<br>49(31.6%)<br>7(4.5%)<br>3(1.9%)	0.567
